# How Does Mg^2+^_(aq)_ Interact with ATP_(aq)_? Biomolecular Structure through the Lens of Liquid-Jet Photoemission Spectroscopy

**DOI:** 10.1021/jacs.4c03174

**Published:** 2024-05-27

**Authors:** Karen Mudryk, Chin Lee, Lukáš Tomaník, Sebastian Malerz, Florian Trinter, Uwe Hergenhahn, Daniel M. Neumark, Petr Slavíček, Stephen Bradforth, Bernd Winter

**Affiliations:** † Fritz-Haber-Institut der Max-Planck-Gesellschaft, Faradayweg 4-6, 14195 Berlin, Germany; ‡ Department of Chemistry, University of California, Berkeley, California 94720, United States; § Chemical Sciences Division, Lawrence Berkeley National Laboratory, Berkeley, California 94720, United States; ∥ Department of Physical Chemistry, University of Chemistry and Technology, Prague, Technická 5, Prague 6 16628, Czech Republic; ⊥ Institut für Kernphysik, Goethe-Universität Frankfurt, Max-von-Laue-Straße 1, 60438 Frankfurt am Main, Germany; # Department of Chemistry, University of Southern California, Los Angeles, California 90089, United States

## Abstract

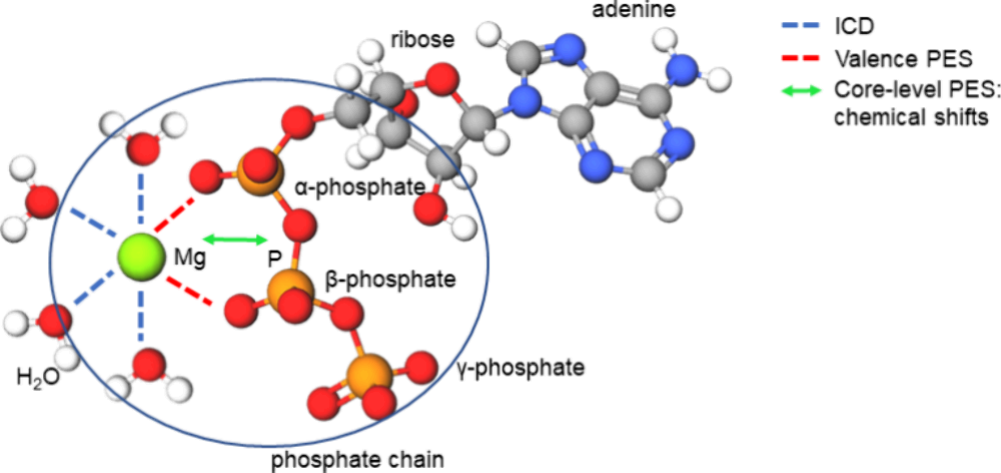

Liquid-jet photoemission spectroscopy (LJ-PES) allows for a direct probing of electronic structure in aqueous solutions. We show the applicability of the approach to biomolecules in a complex environment, exploring site-specific information on the interaction of adenosine triphosphate in the aqueous phase (ATP_(aq)_) with magnesium (Mg^2+^_(aq)_), highlighting the synergy brought about by the simultaneous analysis of different regions in the photoelectron spectrum. In particular, we demonstrate intermolecular Coulombic decay (ICD) spectroscopy as a new and powerful addition to the arsenal of techniques for biomolecular structure investigation. We apply LJ-PES assisted by electronic-structure calculations to study ATP_(aq)_ solutions with and without dissolved Mg^2+^. Valence photoelectron data reveal spectral changes in the phosphate and adenine features of ATP_(aq)_ due to interactions with the divalent cation. Chemical shifts in Mg 2p, Mg 2s, P 2p, and P 2s core-level spectra as a function of the Mg^2+^/ATP concentration ratio are correlated to the formation of [Mg(ATP) _2_]^6–^_(aq)_, [MgATP]^2–^_(aq)_, and [Mg_2_ATP]_(aq)_ complexes, demonstrating the element sensitivity of the technique to Mg^2+^–phosphate interactions. The most direct probe of the intermolecular interactions between ATP_(aq)_ and Mg^2+^_(aq)_ is delivered by the emerging ICD electrons following ionization of Mg 1s electrons. ICD spectra are shown to sensitively probe ligand exchange in the Mg^2+^–ATP_(aq)_ coordination environment. In addition, we report and compare P 2s data from ATP_(aq)_ and adenosine mono- and diphosphate (AMP_(aq)_ and ADP_(aq)_, respectively) solutions, probing the electronic structure of the phosphate chain and the local environment of individual phosphate units in ATP_(aq)_. Our results provide a comprehensive view of the electronic structure of ATP_(aq)_ and Mg^2+^–ATP_(aq)_ complexes relevant to phosphorylation and dephosphorylation reactions that are central to bioenergetics in living organisms.

## 1 Introduction

Photoemission spectroscopy is a method of choice for the characterization of solid-state systems as it directly probes the electronic structure of materials and surfaces.^1^ The application of this powerful tool to aqueous solutions–the main domain of chemistry–has been complicated by the high water vapor pressure inherent to these systems, which had prevented the detection of the photoemitted electrons. However, the advent of the liquid-microjet technology marked a breakthrough.^2^ This advance allows high-quality photoemission spectra to be recorded on an absolute energy scale,^3^ providing novel chemical insights into a number of areas, *e.g*., the electronic structure of liquid water and coordination compounds,^4,5^ ion pairing,^6^ acid–base properties in polyprotic systems,^7^ oxidation of nucleic acids,^8^ or the depth profile of air–water interfaces.^9^ The technique has been so far rarely used in the field of biochemistry, which includes large molecules in complex environments. Here, we demonstrate how liquid-jet photoemission spectroscopy (LJ-PES) assesses specific molecular interactions that can provide structural information for such systems, exemplified here for the buffered adenosine triphosphate nucleotide (ATP_(aq)_).

**Figure 1:**
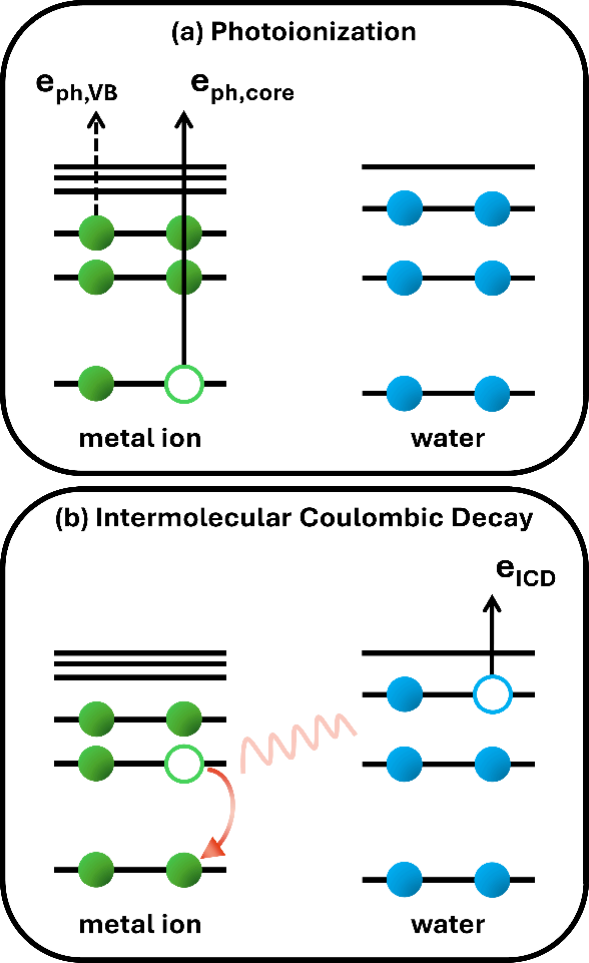
(a) Emitted valence or core-level photoelectrons upon photoionization of the ionic solute in water. (b) Intermolecular Coulombic decay (ICD): A deep core hole relaxes via refill by a valence electron from the same atom, and the released energy is used to ionize the first solvation shell, here depicted for a hydrating water molecule.

The interaction of molecules with soft X-ray photons leads to the photoemission of electrons generated in different processes. Let us begin by inspecting the possible spectral regions that can be recorded in a LJ-PES measurement; see Figure 1(a). The valence electrons are the easiest to ionize; their binding energies (BEs) are observed in the 10–20 eV range for virtually all solvated molecules, including water.^4,10^ This part of the photoemission spectrum bears, therefore, rather limited structural information but it is important for understanding, *e.g*., redox properties.^8^ The core-level electrons (1s) are often used as a sensitive probe of the molecular constitution.^7^ They are energetically well-separated for different atoms, and in addition, their chemical environment controls the so-called “chemical shift”. This spectroscopy is thus analogous to nuclear magnetic resonance (NMR) spectroscopy. The inner-valence photoelectrons (*e.g.*, 2s or 2p) can bear similar type of information, especially for heavier atoms, but this spectral range is less explored. Note that within a single measurement we can obtain spectra associated with all atoms and all energy levels, provided a sufficiently high photon energy is used. We show below that photoelectron (PE) spectroscopy is applicable for disentangling even intermolecular interactions, although the chemical shifts brought about by these interactions are often surprisingly small.^11^

Yet another type of structural information is revealed by second-order electron emission, *i.e.*, the Auger-type electrons. The core hole formed upon ionization can be refilled by an inner-shell valence electron, with the released energy causing autoionization of another valence electron. This is the Auger–Meitner process if it takes place within a single atom, and these electrons are only little affected by chemical environment.

In recent years, much attention has been paid to an analogous type of processes taking place intermolecularly. In intermolecular Coulombic decay (ICD),^12^ the generated core hole is filled by a valence electron, and the excess energy is used to ionize a neighboring molecule; this nonlocal autoionization process is depicted in Figure 1(b). The probability of the process scales with 1/R^6^, where R is the distance between the two neighboring atomic or molecular entities. Hence, ICD uniquely probes the first solvation shell and, equivalently, ion pairing and weak ion binding/association; the process is even sensitive to molecular orientation.^13^ The energy of a specific ICD feature, associated with two locally separated valence holes (open circles in Figure 1(b)), depends on the electronic structure of each entity. ICD can serve as a molecular ruler, in analogy to the fluorescence resonance energy transfer (FRET) process.^14^ In fact, both processes are controlled by the same Coulombic matrix elements, yet ICD is not subject to any strict selection rule.^12,14^ While this application of the ICD process has been hypothesized since its discovery,^12^ to our knowledge, the present work shows ICD’s practical realization for the first time.

**Figure 2:**
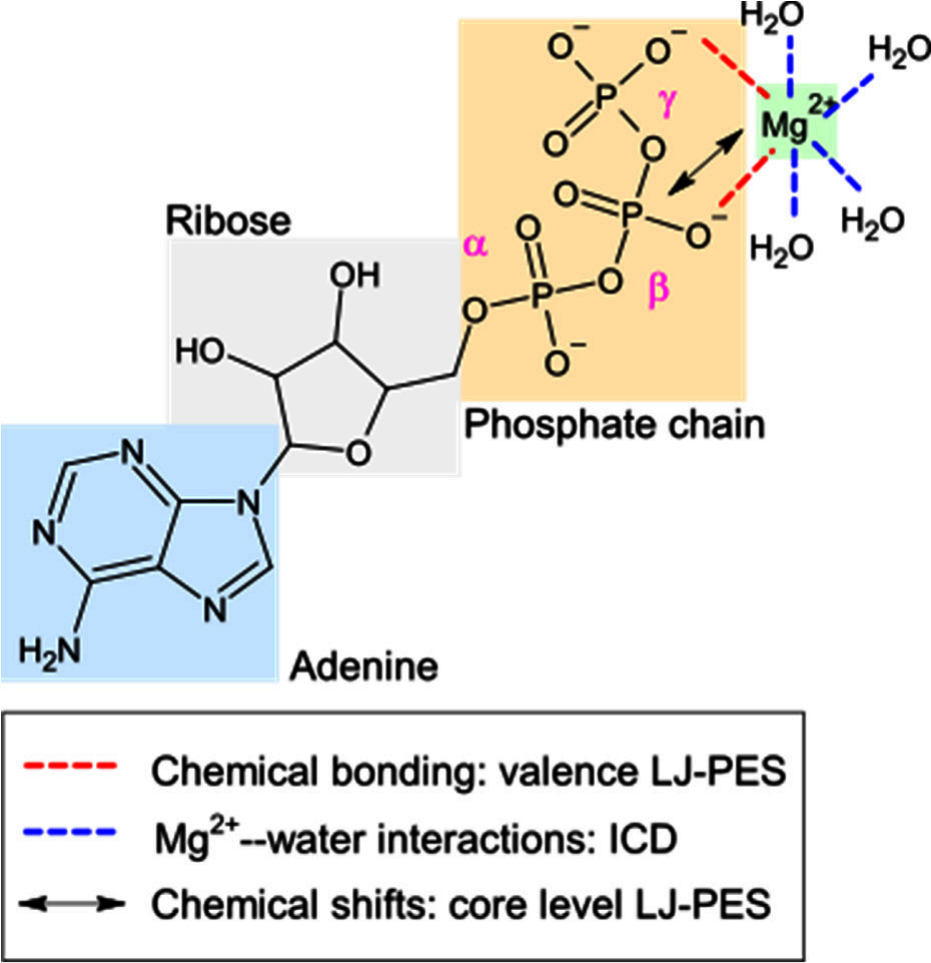
(a) Molecular structure of ATP_(aq)_ in the deprotonated form (ATP^4–^_(aq)_), as predominantly found at physiological pH. Adenine, ribose, and α-, β-, and γ-phosphate units are labeled; one of many motifs for Mg^2+^ binding is shown (see text). Molecular interactions probed using valence and core-level LJ-PES, as well as ICD spectroscopy, are highlighted using red and blue dashed lines, respectively.

ATP_(aq)_ consists of a nucleoside (adenosine, which is formed by adenine and ribose) bound to a chain of three phosphate groups^15^ that enables energy exchange and signal transduction in living organisms.^16−21^ ATP’s molecular structure is illustrated in Figure 2, highlighting the adenine, ribose, and phosphate units. The latter are designated as α, β, and γ (where α refers to the phosphate directly bound to the nucleoside, β refers to the bridging phosphate unit, and γ refers to the terminal phosphate) and are shown in their deprotonated form (ATP^4–^_(aq)_, as predominantly found at physiological pH).^22^ We also include Mg^2+^_(aq)_, or more specifically, [Mg(H_2_O)_6_]^2+^_(aq)_, the octahedral complex configuration adopted by the free ion in aqueous solution.^23^ This ion interacts with one or more phosphate units to form various Mg^2+^–ATP_(aq)_ complexes,^24−26^ such as [Mg(ATP)_2_]^6–^_(aq)_,^24,27−30^ Mg_2_ATP_(aq)_,^31^ and [Mg_2_(ATP)_2_]^4–^_(aq)_.^24,32^ For [MgATP]^2–^_(aq)_, a closed form has also been proposed, where the metal ion interacts not only with O atoms from the phosphate units but also, presumably through a water molecule, with a N atom from the adenine unit. The “closed form”/“open form” ratio was reported to be 1:10 in aqueous solution.^21,33−36^ A schematic representation of this configuration is shown in Figure S1 in the Supporting Information (SI).

The biological function of ATP_(aq)_ stems from the liberation and addition of phosphate units, termed dephosphorylation and phosphorylation reactions, respectively, in the aqueous environment of the cell.^37^ In the former case, energy is released via hydrolysis to produce adenosine diphosphate (ADP), and subsequent hydration of the latter. In the latter case, phosphorylation of ADP_(aq)_ takes place to restore ATP_(aq)_ in the cell. In this way, metabolic pathways involving ATP_(aq)_ and ADP_(aq)_ are regulated by chemical bond breaking and bond formation at the phosphate chain.^38^ Despite a single phosphate unit seemingly being involved in phosphorylation and dephosphorylation reactions,^39^ Mg^2+^_(aq)_ complexation to multiple sites in the phosphate chain is required for the overall reaction to proceed.^40−43^ In the absence of enzymes, α-, β-, and γ-phosphate are expected to be equally involved in Mg^2+^_(aq)_ association to ATP_(aq)_.^26,44,45^ In enzyme-bound ATP_(aq)_, the β- and γ-phosphate units are most likely to interact with Mg^2+^_(aq)_.^46^ The binding constants associated with specific Mg^2+^–ATP_(aq)_ ion pairing motifs alter the Gibbs free energy of hydrolysis,^47^ as they determine the concentration of each of the species involved in the reaction.

Overall, phosphorylation and dephosphorylation processes are strongly affected by the presence of divalent metal cations, particularly by Mg^2+^_(aq)_, the cation with the highest ATP_(aq)_ binding affinity,^25,34^ and by hydration effects inherent to the aqueous environment of the cell.^46,48−50^ In this way, metal–ligand coordination at the phosphate chain is involved in dephosphorylation (hydrolysis) or phosphorylation at specific phosphate units via charge redistribution and conformational changes,^24,46^ with the relatively small size of the Mg^2+^_(aq)_ ion facilitating coordination.^51^ These bonding interactions should result in charge redistribution at the P–O–P bond^39^ and differences in electron BEs of the α-, β-, and γ-phosphates in ATP_(aq)_ and Mg^2+^–ATP_(aq)_. Such intramolecular (and intermolecular) charge redistributions, as well as solvation effects, are reflected in the phosphorylation and dephosphorylation reaction mechanisms.^39,40,43,52^ The nucleobase–metal-ion interactions mentioned earlier may also play a role in the overall reaction.^53^ A large amount of association equilibria data has been determined in support of such effects.^54^ However, the characterization of associated structures by advanced spectroscopic methods is lacking.

With that in mind, we aim to investigate whether LJ-PES,^10,55^ which has been used to probe chemical shifts in inorganic and organic solutes in aqueous environments^7,8,56−58^ is (1) sufficiently sensitive to ATP_(aq)_ ion pairing and/or complexation, and (2) capable of distinguishing between the three phosphate groups. Regarding the first question, we report on the electronic-structure changes upon the addition of Mg^2+^ to ATP_(aq)_ solutions and the formation of different Mg^2+^–ATP_(aq)_ complexes, inferred from valence and Mg 2p, Mg 2s, P 2p, and P 2s core-level PE spectra. To elaborate on the second, we also present P 2s spectra from aqueous solutions of neat ATP_(aq)_, ADP_(aq)_, and AMP_(aq)_, determining relative changes in the BEs of α-, β-, and γ-phosphate.

We contrast the (direct) PE spectra (compare Figure 1(a)) with nonlocal autoionization electron signal, upon ICD^5,13,14,59,60^ of Mg^2+^_(aq)_ ions (compare Figure 1(b)) in the presence of ATP_(aq)_ in their coordination environment. The unique sensitivity to the first hydration shell has been recently demonstrated for Mg^2+^ in water^13^ which lays the groundwork for the present study. The detected ICD electron signal senses the interaction of the metal ion with its immediate neighbor in an exclusive and most direct way, namely by the selective autoionization of just the constituent of the first solvation shell. To be more specific, we measure the ICD electrons formed upon photoionization of Mg 1s core-level electrons. Here, the respective core hole is refilled by electrons from the Mg 2s (or Mg 2p) core levels, and the released excess energy is used to ionize the surrounding molecules in the first solvation shell. The emitted second-order electron is then detected as an ICD signal; to be detailed below.

Ideally, the experiments could be interpreted without the need of *ab initio* theory. The changes of both the PE and ICD signals can indeed be generally interpreted within simple electrostatic concepts. However, these views can be oversimplified, and it is, therefore, still desirable to confirm the experiments with *ab initio* calculations. These calculations help to understand the quantitative correlations in the experiments.

Due to the importance of ATP_(aq)_ in biochemistry, the Mg^2+^–phosphate interactions have been previously explored by many techniques, *e.g.*, using aqueous-phase X-ray emission,^61^ infrared,^36^ Raman,^62^ and nuclear magnetic resonance (NMR)^63^ spectroscopies. The Mg^2+^–ATP coordination chemistry has been recently investigated in the gas phase using mass spectrometry (in particular, phosphate–Mg^2+^–adenine interactions)^35^ and in acetate solutions^64^ using NMR spectroscopy. Previous LJ-PES work focused on the ribose or adenine units in ATP_(aq)_,^65−67^ or on inorganic phosphate^6^ aqueous solutions. The present LJ-PES results can thus be compared to these techniques, demonstrating its scope of applicability.

## 2 Methods

### 2.1 Experiments

0.5 M ATP_(aq)_, ADP_(aq)_, and AMP_(aq)_ solutions were prepared by dissolving the required amount of adenosine 5′-triphosphate disodium salt hydrate, adenosine 5′-diphosphate acid, and adenosine 5′-monophosphate disodium salt (Carbosynth, 95%) in Millipore water, respectively. ATP_(aq)_ and ADP_(aq)_ samples containing Mg^2+^ were prepared by addition of Mg(NO_3_)_2_ (Acros Organics, 99+%) to 0.5 M ATP_(aq)_ or ADP_(aq)_ solutions in order to reach 0.25:1, 0.5:1, 0.75:1, 1:1, and 1.5:1 Mg^2+^/ATP concentration ratios. For each sample, the solution pH was adjusted to 8.2 by the addition of the required amount of Tris (tris(hydroxymethyl)aminomethane, Sigma-Aldrich, ≥ 99.8%), to ensure that the ATP_(aq)_ phosphate chain was fully deprotonated (ATP^4–^_(aq)_, see Figure 2).^22,27,68^ This alternative approach to the traditional Tris/TrisHCl buffer pH adjustment methodology allows us to reduce the number of chemical species in the solution that can interfere with the observation of the PE signals of interest. The specific concentration of Tris_(aq)_ in each sample is listed in Table S1 in the SI. The Mg 2s core-level PE spectra from a 0.5 M Mg(NO_3_)_2(aq)_ solution with a pH adjusted to 8.4 was measured for reference.

LJ-PES experiments were performed at the P04 beamline at PETRA III^69^ (DESY, Hamburg, Germany) using the EASI setup.^70^ Photoelectrons emitted from the sample were detected using a differentially pumped hemispherical electron analyzer at 130° with respect to the light propagation axis (circular polarization). The samples were delivered into the vacuum chamber of the EASI setup in the form of liquid microjets^71^ using a glass capillary of 28 μm inner diameter and flow rates in the range of 0.55–0.80 mL/min. The sample temperature was kept at 10 °C by means of a cooling system interfaced with the liquid-jet holder. A small metallic wire was placed into the main polyether ether ketone (PEEK) liquid delivery line to electrically connect and ground the liquid jet to EASI. The liquid jet’s (horizontal) flow axis was perpendicular to both the light propagation (floor plane) and the electron detection (at an angle of 130° with respect to the photon beam) axes. PE spectra from ATP_(aq)_ with dissolved Mg^2+^ were recorded using a photon energy of 250 eV, spanning over the valence, Mg 2p, Mg 2s, P 2p, and P 2s spectral regions. To determine core-level chemical shifts with better precision, additional PE spectra focused on measuring only the Mg 2s, P 2p, and P 2s regions with finer step width (0.05 instead of 0.2 eV) were recorded at 250 eV, 270 eV, and 330 eV photon energy, respectively. Immediately before or after each of these a spectrum of the water valence region was measured with all other parameters identical, to aid in BE calibration. P 2s PE spectra from AMP_(aq)_, ADP_(aq)_, and ATP_(aq)_ were also recorded, using a photon energy of 330 eV. ICD experiments were performed at 1314 eV. The overall instrumental energy resolution was approximately 210 meV at 250 eV, 220 meV at 330 eV, and 570 meV at 1314 eV photon energy.

The BE scale in the data presented here was calibrated based on the liquid water 1b_1_ BE of 11.33 eV,^3^ as commonly adopted in LJ-PES experiments.^11^ While we have recently reported a more robust methodology for determining absolute aqueous-phase electron BEs^3^ (see note above), the data acquisition of the results presented here preceded those developments. However, absolute BEs are not the principal quantity of interest in this work, but rather relative spacings of peaks that can be attributed to different species in solution.

### 2.2 Computations

α-, β-, and γ-phosphate P 2s BEs of ATP^4–^_(aq)_, [Mg(ATP)_2_]^6–^_(aq)_, [MgATP]^2–^_(aq)_, Mg_2_ATP_(aq)_, and [MgADP]^−^_(aq)_ were calculated using the maximum-overlap method (MOM)^72^ as implemented in the Q-Chem 6.0 software^73^ (the sample input for these calculations can be found in the SI). An excellent computational cost/performance ratio was previously demonstrated with this approach.^74^ Due to the system size, we pragmatically employed the Hartree–Fock (HF) method with a core-enhanced aug-cc-pCVTZ basis set on P atoms and aug-cc-pVTZ basis set on other atoms. To model Mg 2s and 2p BEs, a core-enhanced aug-cc-pCVTZ basis set was used for Mg atoms and aug-cc-pVTZ for other atoms. The aqueous solution was modeled by the cluster–continuum approach.

For our quantum system, we explicitly included 26 water molecules around the triphosphate chain (to cover each terminal O with at least three hydrogen bonds from water molecules) of ATP^4–^_(aq)_, [MgATP]^2–^_(aq)_, and Mg_2_ATP_(aq)_, 17 water molecules for [MgADP]^−^_(aq)_, and 44 water molecules for [Mg(ATP)_2_]^6–^_(aq)_ to screen the high charge density while the rest of the solvent was described by the polarizable-continuum model (PCM).^75,76^ We used the nonequilibrium variant of PCM with integral-equation formalism (IEF), Bondi radii, and recommended scaling factor α = 1.2.^77^ Note that calculations of multiply charged ions are known to have limitations^6^ as, *e.g.*, including extensive explicit solvation shell or counterions might be required. Consequently, our computed BEs will only provide a framework for the spectra interpretation rather than precise values. For pure Mg^2+^_(aq)_ solutions, the cation was solvated by six explicit water molecules in an octahedral geometry, while the rest of the solvent was modeled by PCM.

The calculated P 2s BEs were corrected for the missing electronic-correlation description in the HF method as follows. We used pyrophosphate (HP_2_O_7_^2–^) hydrated by five explicit water molecules as a smaller model of the phosphate chain. We computed the P 2s BEs using the HF and the second-order Møller–Plesset (MP2) methods to approximately determine the error connected to the missing electronic correlation in the HF method. The correction was estimated to be +0.33 eV. The calculations of BEs were done on single structures optimized on the HF/6-31+G* level of theory with PCM (IEF, Bondi radii, α = 1.2) using Gaussian 09, revision D.01^78^ (the Cartesian coordinates can be found in the SI). Due to its size, [Mg(ATP)_2_]^6–^_(aq)_ with 44 water molecules could not be fully optimized and the structure obtained after 144 optimization steps was used.

The valence-region BEs of HOMO, HOMO–1, and HOMO–2 were calculated using Δ*S*CF and MOM, respectively, with the CAM-B3LYP functional^79^ and the aug-cc-pVTZ basis set. The solvation was modeled by including 5 explicit water molecules (three around the phosphate chain and two around the adenine moiety) and PCM (IEF, Bondi radii, α = 1.2). The calculations were performed for two optimized structures, one for the ATP molecule without the Mg ion and one corresponding to the “open form” of [MgATP]^2−^_(aq)_. The optimization of structures was done on the CAM-B3LYP/6-31+G* level of theory, according to our previous work.^7^ The Cartesian coordinates of the optimized structures can be found in the SI.

## 3 Results and Discussion

### 3.1 Mg^2+^–ATP_(aq)_ Interaction: Perspective of Element-Specific Photoelectron Spectra

This section presents valence and core-level PE spectra from ATP_(aq)_ samples with dissolved Mg^2+^, providing an overview of the electronic structure of ATP_(aq)_ and the interactions between the metal cation and different units in the ATP_(aq)_ molecule.

**Figure 3:**
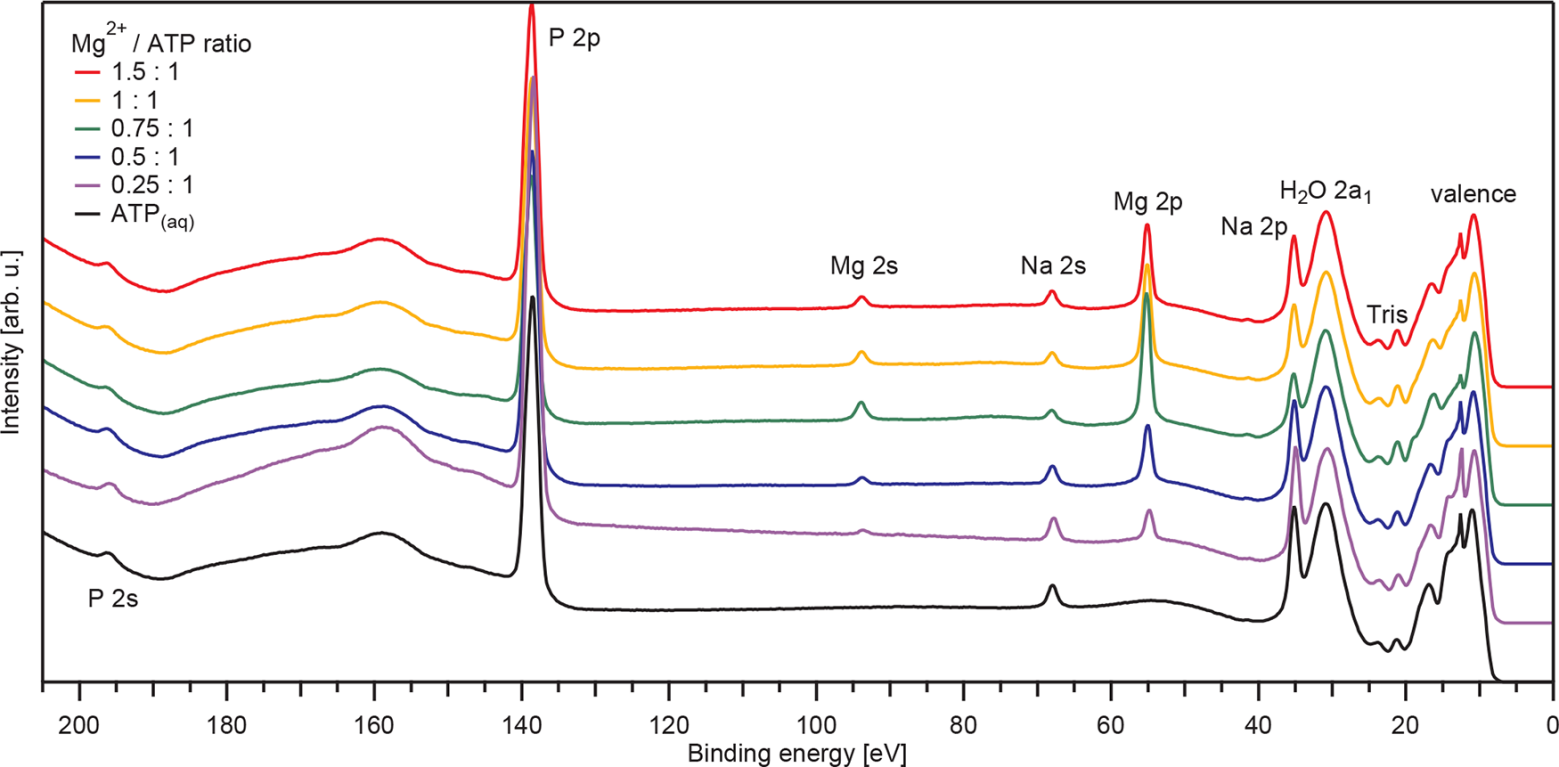
Valence as well as Mg 2p, Mg 2s, P 2p, and P 2s core-level PE spectra from ATP_(aq)_ samples containing Mg^2+^_(aq)_ as a function of the Mg^2+^/ATP concentration ratio, measured at 250 eV photon energy. The leading peaks are from ionization of the water 1b_1_ orbital. The bottom spectrum is from an ATP_(aq)_ solution without added Mg^2+^_(aq)_.

**Figure 4:**
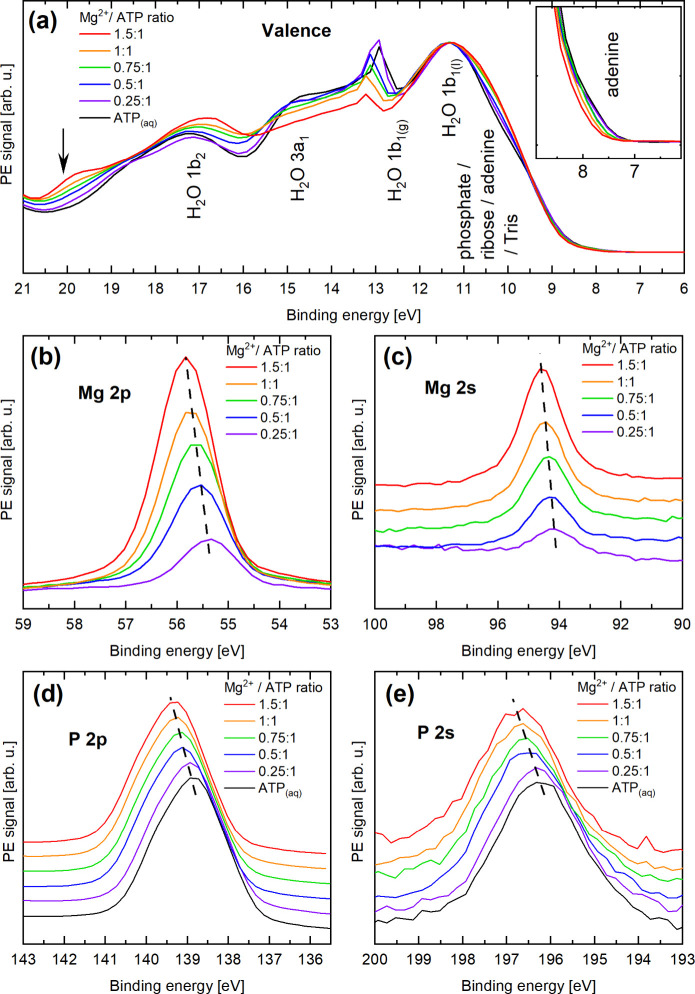
Highlights of the valence [panel (a)], Mg 2p [panel (b)], Mg 2s [panel (c)], P 2p [panel (d)], and P 2s [panel (e)] spectral regions from the data presented in Figure 3. In panel (a), PE signatures of the ionization of water valence electrons are labeled as (liquid- and gas-phase) 1b_1_, 3a_1_, and 1b_2_, according to ref (4). The arrow highlights PE signatures due to Mg^2+^–phosphate interactions, as explained in the text. The Mg 2s, P 2p, and P 2s data are shown with a vertical offset for a better comparison, and chemical shifts are highlighted by the dashed lines. The P 2p and P 2s data were normalized in intensity, considering that the ATP_(aq)_ concentration remains constant across those data sets (as opposed to the Mg^2+^_(aq)_ concentration in the Mg 2p and Mg 2s spectra, whose variation is reflected in the peak intensities). Linear baselines were subtracted from the P 2s data to remove the secondary electron background (see Figure S5 in the SI for details).

PE spectra from 0.5 M ATP_(aq)_ solutions with Mg^2+^_(aq)_ at different Mg^2+^/ATP concentration ratios recorded at a photon energy of 250 eV are shown in Figure 3. The displayed wide energy range covers ionization of the valence band, and the Mg 2p, Mg 2s, P 2p, and P 2s core levels. For reference, the respective spectrum recorded from 0.5 M ATP_(aq)_ without Mg^2+^_(aq)_ is also shown. The as-measured spectra were energy calibrated as described in the Methods section, and intensities were normalized to yield the same peak height as that of the liquid-water lowest ionization feature, 1b_1_. Spectral features from the Na^+^ counterion in the ATP salt used to prepare the solutions are labeled according to ref (81). The intensity of this feature is observed to decrease as the Mg^2+^_(aq)_ concentration increases, which might be due to changing propensity of the solute under study at the surface. Contributions from the NO_3_^–^_(aq)_ counterion from the Mg^2+^ salt used in the experiments are expected in the 9.0–9.5 eV BE range.^10^ Contributions from Tris added to adjust the pH are labeled based on ref (8). Valence PE data from Tris_(aq)_ solutions is shown in Figure S2 in the SI, along with a discussion of its potential effect on spectral shifts. A zoom into the different spectral regions is shown in Figure 4 [panels (a)-(e)].

Figure 4(a) expands the valence region, which is dominated by PE peaks arising from (direct) ionization of water valence electrons,^4^ labeled as (liquid- and gas-phase) 1b_1_, 3a_1_, 1b_2_, and 2a_1_ (the latter only included in Figure 3). Observed small energy shifts and intensity variations of the 1b_1(g)_ gas-phase peak, from the surrounding water vapor, result from the combined effects of change of solution surface potential and small changes of water electronic structure upon addition of solute.^3,81^ Based on a previous valence LJ-PES study of AMP_(aq)_^8^ and valence PE spectra from ADP_(aq)_ and ATP_(aq)_ (see Figure S3 in the SI for details), the phosphate, ribose, and adenine PE signatures contribute in the 8–10 eV BE range, with an additional adenine feature also present at 7–8 eV, as highlighted in the inset of Figure 4(a). The latter peak is observed to shift as the Mg^2+^/ATP concentration ratio increases. Similar shifts are observed in the valence PE spectra recorded from ADP_(aq)_ solutions as a function of the Mg^2+^/ADP concentration ratio (see Figure S4 in the SI). These energy shifts for ATP are in qualitative agreement with our *ab initio* calculations. As shown in Table 1, the low-energy signal is comprised of the ionization of adenine’s highest occupied molecular orbital (HOMO), as well as HOMO–1 and HOMO–2. It is seen that on average, the BEs are larger in the presence of Mg^2+^.

**Table 1:** Calculated Valence BEs (in eV) for ATP and ATP–Mg structures

	HOMO	HOMO–1	HOMO–2
ATP without Mg	7.47	7.69	7.99
ATP–Mg	7.40	8.18	8.43

Figure 4(a) reveals further Mg^2+^-concentration-dependent spectral changes, in the 10–20 eV BE range, which can be also associated with the ionization of adenine and ribose orbitals, at lower BEs, and phosphate chain at higher energies.^82^ Most noticeable is the emerging 20 eV BE peak (see the arrow) upon increasing the Mg^2+^/ATP concentration ratio which can be attributed to the presence of Mg^2+^–phosphate interactions in any of the Mg^2+^–ATP_(aq)_ moieties in which O atoms from the phosphate chain are directly exposed to the positive charge from the divalent metal cation. Previous LJ-PES experiments have been shown to be sensitive to Na^+^–phosphate electrostatic interactions,^6^ and we expect the presence of Mg^2+^_(aq)_ to have a more pronounced effect. To confirm our assignment, we have performed theoretical calculations employing the same methodology used to calculate P 2s BEs as detailed in the Computations section. Our results indicate that the 20 eV BE feature originates from ionization of electrons from mixed, delocalized molecular orbitals appearing when Mg^2+^_(aq)_ ions are closely bound to the phosphate chain, with significant contributions from O, C, and P atoms. We note that O 2s signatures from phosphate compounds are expected in the 24.0–25.7 eV BE range,^83^ and are likely hidden underneath the water 2a_1_ peak. Another striking spectral change when increasing the Mg^2+^/ATP concentration ratio is the larger intensity on the low-BE side of the water 1b_1_ peak, occurring approximately between 9.5–11.0 eV BE. As we have shown in a previous work,^8^ this region contains contributions from phosphate, centered near 9 eV BE, and ribose contributions, around 10 eV.

**Figure 5:**
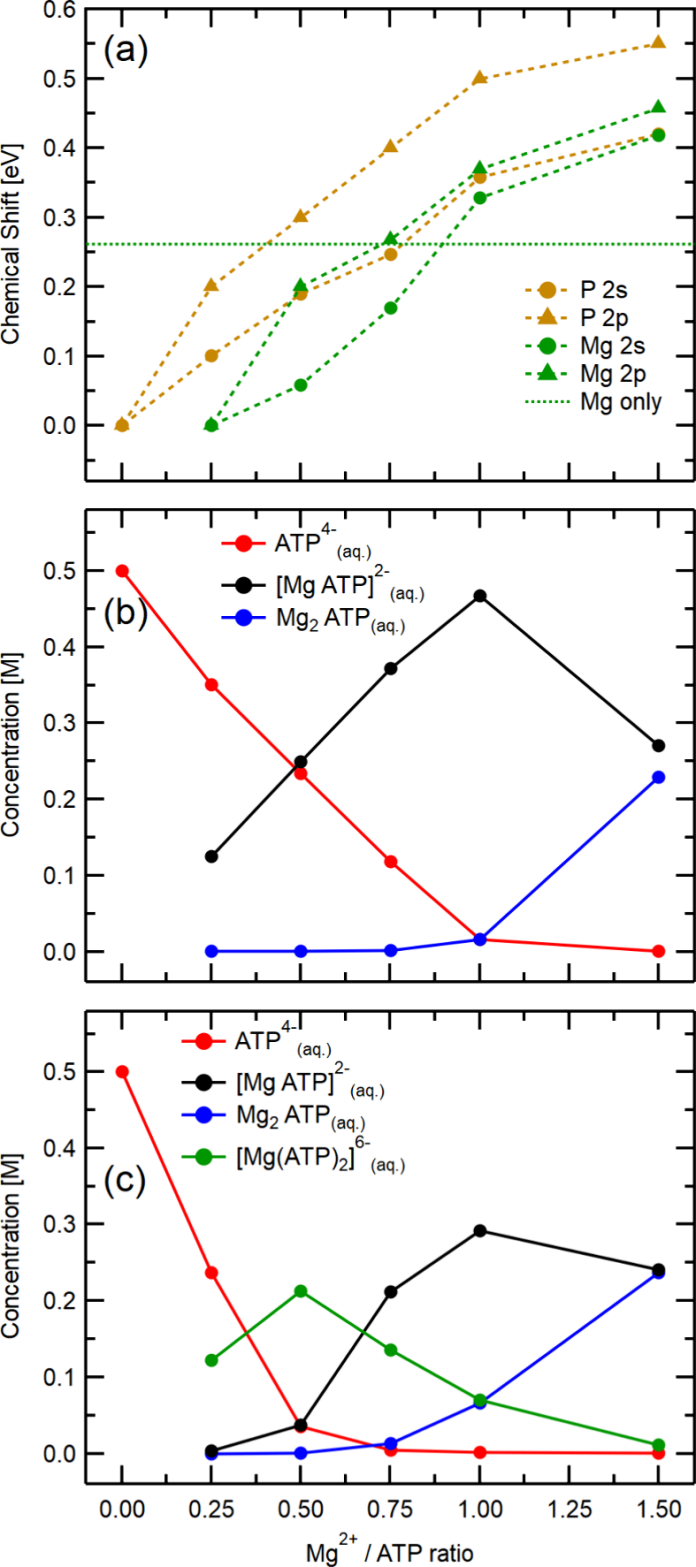
(a) Chemical shifts extracted from measurements of the Mg 2p, Mg 2s, P 2p, and P 2s core levels as a function of the Mg^2+^/ATP concentration ratio. The Mg 2s value measured for a 0.5 M Mg(NO_3_)_2(aq)_ solution without ATP is shown by the green-dotted line for reference. (b) and (c) Molar concentration of the predominant species ATP^4–^_(aq)_, [MgATP]^2–^_(aq)_, Mg_2_ATP_(aq)_, and [Mg(ATP)_2_]^6–^_(aq)_ as a function of the Mg^2+^/ATP concentration ratio at a solution pH of 8.2, calculated within this work using equilibrium constants collected in refs (25) and (28). Details on the two procedures can be found in the SI.

More specific information on Mg^2+^–ATP interaction is retrieved from the core-level spectra. For samples containing Mg^2+^_(aq)_, the Mg 2p and Mg 2s core-level photoelectron signal occurs near 55 and 95 eV, and is highlighted in Figures 4(b) and 4(c), respectively. With increasing Mg^2+^/ATP concentration ratio, the respective intensities increase, and the peak centers shift to higher BEs; these shifts are indicated by dashed lines. We observe similar trends in the P 2p and P 2s spectral regions, near 140 and 196 eV BEs, as highlighted in Figures 4(d) and 4(e). All observed chemical shifts are plotted in Figure 5(a). These shifts can be qualitatively assigned to a change of the population of different Mg^2+^−ATP species, as we detail later.

We start the discussion of the core-level spectra with a comment on the solution composition of the Mg^2+^/ATP_(aq)_ samples studied here using the binding constants collected in ref (27) and the calculation of different Mg^2+^–ATP_(aq)_ complexes reported there (see the SI for details, including Table S2). While these values refer to lower ionic strength conditions compared to the samples studied in this work, our primary focus is to produce a first-order approximation speciation plot to guide our qualitative description of the data. Using the consistent data set of equilibrium constants, ATP^4–^_(aq)_ and [MgATP]^2–^_(aq)_ are characterized as the dominant species at Mg^2+^/ATP concentration ratios between 0.25:1 and 0.75:1, with an increasing proportion of [MgATP]^2–^_(aq)_ as the concentration of Mg^2+^ increases. At a 1:1 Mg^2+^/ATP ratio, there is no longer free ATP_(aq)_ (ATP^4–^_(aq)_) in solution, and [MgATP]^2–^_(aq)_ is the prevailing species. At a 1.5:1 Mg^2+^/ATP ratio, the [MgATP]^2–^_(aq)_ concentration decreases as the formation of [Mg_2_ATP]_(aq)_ becomes relevant. We remind the reader that the amount of free Mg^2+^_(aq)_ is negligible at all the Mg^2+^/ATP concentration ratios studied here. The results are summarized in Figure 5(b).

We consider this a minimal set of species expected in significant amounts in the prepared solutions as suggested by the speciation analysis of ref (27). However, based on subsequently observed^25^ Mg NMR peak broadening for solutions with [ATP] > [Mg^2+^], Bock et al.^28^ concluded that an additional species must be taken into account, in the case of similar or higher [ATP]/[Mg^2+^]. Using apparent association constants from that work, we calculated an extended speciation plot that includes [Mg(ATP)_2_]^6–^_(aq)_; see Figure 5(c). The picture is now somewhat different from our initial figure, with the [Mg(ATP)_2_]^6–^_(aq)_ species being dominant for low Mg^2+^ concentrations.^23^ We will show that measured chemical shifts can indeed be used to probe speciation, and we can in particular determine which of the two reported models better matches the experiment.

The different species have different manifestations in the PE spectrum. Based on simple electrostatic reasoning, we could expect an electron stabilization (increase of the BE) for the electrons in the P atom upon complexation with Mg^2+^. This is generally confirmed by our P 2s calculations presented in Table 3 in the following section. On the other hand, the electrons in Mg^2+^ should be destabilized by the interaction with the negative charge of ATP_(aq)_. To confirm the expected trends, we performed calculations of Mg 2s and Mg 2p BEs for different species. The results are presented in Table 2. Our computations reveal a decrease in BEs when moving from pure Mg^2+^_(aq)_ to [MgATP]^2–^_(aq)_ and an increase in BE when moving to [Mg_2_ATP]_(aq)_. The predicted effect on chemical shifts is approximately the same for Mg 2s and Mg 2p.

**Table 2:** Calculated Mg^2+^_(aq)_ and Mg^2+^–ATP_(aq)_ Mg 2s and Mg 2p BEs (in eV)

	Mg 2s	Mg 2p
Mg^2+^_(aq)_	96.01	55.24
[Mg(ATP)_2_]^6–^_(aq)_	94.03	53.27
[MgATP]^2–^_(aq)_	95.21	54.42
[Mg_2_ATP]_(aq)_	96.08	55.30

This is in qualitative, yet to be detailed, agreement with the trend observed for the Mg 2s data in Figure 5(a). Core-level BEs were determined from Voigt fits with a cubic background (Mg 2s, Mg 2p, and P 2s), or from reading the positions of the peak maxima (P 2p). For Mg 2s and P, this figure was compiled from short-range scans of the core levels, as detailed in the Methods section. For the Mg^2+^_(aq)_-only solution, our calibrated BE (see green-dotted horizontal line) is in good agreement with a measurement of a 3 M MgCl_2(aq)_ solution reported earlier (94.47 versus 94.50 eV),^84^ if the liquid water 1b_1_ BE used in that work is set to 11.33 eV, the value used here. Generally, however, we must expect an inherent experimental error in determining accurate absolute BEs when calibrating to peak positions measured from neat liquid water, as we are not accounting for solute-induced effects on the water electronic structure. As we detail in the SI the magnitude of the associated spectral shifts is, however, typically smaller than the variations seen in Figure 5(a). Although uncertainties in the absolute BEs are thus up to 200 meV, the individual error in each data point on the relative scale used in the figure is below 0.1 eV (±0.05 eV).

We can now test the observed concentration dependence against the aforementioned speciation models, Figures 5(b) and 5(c). Following the Occam’s razor principle, we will start with the minimal model, assuming the [MgATP]^2–^_(aq)_ species is the totally dominating form up to the 1:1 Mg^2+^/ATP concentration ratio (Figure 5(b)). The P core-level BE should then increase monotonically with concentration. This is indeed the case (see Figure 5(a)); the signal exhibits a clear positive chemical shift (*i.e.*, toward higher BEs), up to 500 meV for the highest Mg^2+^/ATP concentration ratios. The Mg core-level BE should drop when moving from Mg^2+^ in water to the complex (data points below the green dashed line), and then it should stay constant. While we see the initial drop, the Mg core-level BEs increase with almost the same slope as the P core-level data.

We conclude that more species are involved in the solutions, consistent with the NMR measurements by Bock et al.^28^ Indeed, the extended speciation plot in Figure 5(c) is in qualitative agreement with the observed chemical shifts. As before, the P core-level BE increases for [MgATP]^2–^_(aq)_ and [Mg_2_ATP]_(aq)_ (corresponding to higher Mg^2+^/ATP concentration ratios). However, there is no predicted shift between ATP^4–^_(aq)_ and the new species [Mg(ATP)_2_]^6–^_(aq)_ (corresponding to lower Mg^2+^/ATP ratios) in Table 3, while it is observed experimentally in Figure 5(a). Nevertheless, such computational discrepancies can be expected as we are comparing the two species with the highest negative charges and the calculations do not include the Na^+^ counterion, affecting the BEs of electrons in the phosphate chain.^6^ Since there is a mixture of these species with free ATP_(aq)_, the shift is gradual.

**Table 3:** Calculated ATP_(aq)_ and Mg^2+^–ATP_(aq)_ P 2s α-, β-, and γ-Phosphate BEs (in eV)

	α	β	γ
[ATP]^4–^_(aq)_	196.86	196.85	195.98
[Mg(ATP)_2_]^6–^_(aq)_	196.98	196.78	195.94
[MgATP]^2–^_(aq)_ (Mg^2+^-bonding to α-, β-, and γ-phosphate)	197.33	197.15	196.31
[MgATP]^2–^_(aq)_ (Mg^2+^-bonding to β- and γ-phosphate)	197.28	197.01	196.39
[Mg_2_ATP]_(aq)_	198.27	197.83	196.82

On the other hand, the shifting of the Mg core-level peaks is even more interesting. The [Mg(ATP)_2_]^6–^_(aq)_ species, formed at the lowest concentration, leads to the destabilization of the Mg^2+^ electrons when compared to the Mg^2+^ ion in water. As seen in Table 2, the destabilization is smaller for [MgATP]^2–^_(aq),_ which is prevalent at higher concentrations, and the BE further grows for [Mg_2_ATP]_(aq)_. Our *ab initio* calculations reveal, however, certain quantitative differences compared to the experiment: the BE destabilization of Mg^2+^_(aq)_ upon complexation with one or two ATP_(aq)_ units is predicted to be larger (800 and 2000 meV) than what is observed (350 and 440 meV), and the chemical shift for [Mg_2_ATP]_(aq)_ is predicted to be too small. In both cases, the Mg^2+^_(aq)_ electrons are over stabilized which could be attributed to the presence of Na^+^ ions (not covered in the calculations) and the computational limitations, describing solvation of highly charged anions with the dielectric continuum model and using only the Hartee–Fock electronic structure theory due to the size of the system.

### 3.2 α-, β-, and γ-Phosphate-Specific Interactions in ATP_(aq)_ and Mg^2+^–ATP_(aq)_

In the previous section we have inspected global chemical shifts, P 2s and P 2p. We now explore whether we can decompose the P core-level peak shape to distinguish between contributions between α-, β-, and γ-phosphate.

As a starting point, we performed computations to calculate the P 2s BEs of each phosphate unit in fully deprotonated ATP_(aq)_ (ATP^4–^_(aq)_), as found at the solution pH of the samples studied here. We obtained nearly identical α- and β-phosphate P 2s BEs (196.86 and 196.85 eV, respectively), and an ∼880 meV lower γ-phosphate P 2s BE (195.98 eV). In agreement with a previous report on P 2p PES from solid-phase calcium tripolyphosphate,^85^ our results show that the bridging phosphate groups (α and β units) have higher BEs than the terminal phosphate (γ-phosphate). In ATP^4–^_(aq)_, P core-level electrons in the γ-phosphate are uniquely subjected to repulsive interactions from two negatively charged O sites, while α and β units contain a single deprotonated site [see Figure 1(a)]. Such differences in the local chemical environment lead to lower γ-phosphate P 2s BE values compared to the α and β units. (We note that the present and following discussions do not extend to P 2p BEs since the expected doublet peak structure would prevent a similarly accurate distinction of the small energetic differences associated with the specific phosphate groups.)

Having computationally explored the magnitude of the differences in BE between each phosphate unit in ATP_(aq)_, we attempted to deconvolve P 2s PE spectra recorded from ATP_(aq)_ samples into individual α-, β-, and γ-phosphate contributions with the aid of spectra from AMP_(aq)_ and ADP_(aq)_. When discussing the respective electron BEs, it is useful to refer to each phosphate group as adenosine–phosphate bridging unit, phosphate–phosphate bridging unit, and terminal unit, rather than α, β, and γ, respectively; this characterization most directly reflects the origin of the different BEs.

**Figure 6:**
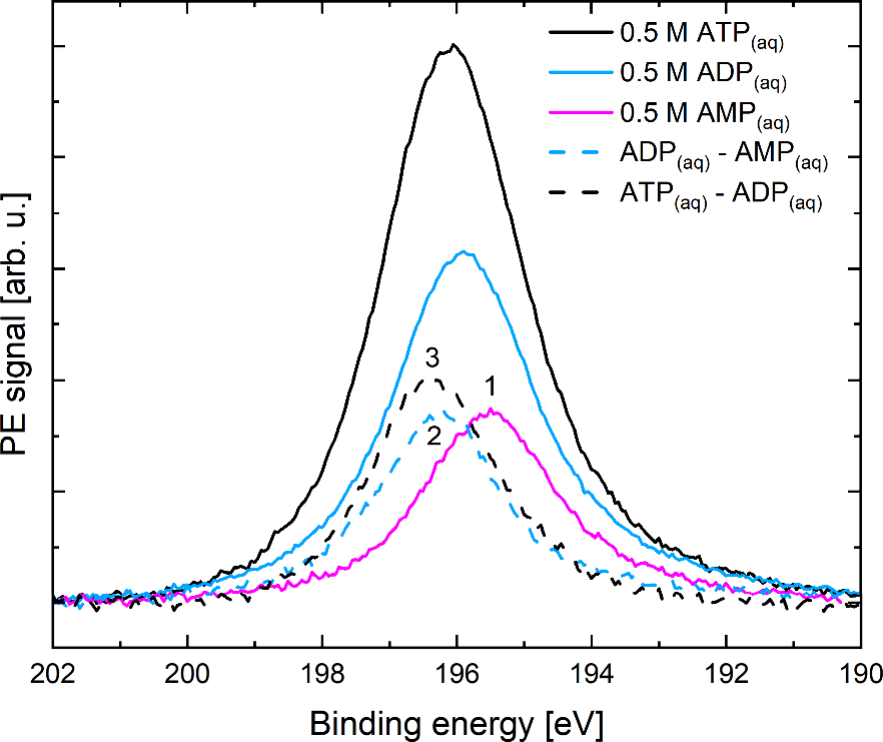
P 2s PE spectra recorded from AMP_(aq)_, ADP_(aq)_, and ATP_(aq)_ solutions without dissolved Mg^2+^. The AMP_(aq)_ data is representative of a terminal phosphate (peak 1), while bridging phosphate units in ATP_(aq)_ (peaks 2 and 3) were identified by subtracting the AMP_(aq)_ data from the ADP_(aq)_ spectrum (cyan dashed line) and the ADP_(aq)_ data from the ATP_(aq)_ spectrum (black dashed line).

Figure 6 shows P 2s PE spectra from 0.5 M AMP_(aq)_ (magenta curve), ADP_(aq)_ (cyan curve), and ATP_(aq)_ (black curve) solutions at pH 8.2 recorded using a photon energy of 330 eV. A linear baseline was subtracted from each spectrum, and the BE scale was calibrated with respect to the liquid-water 1b_1_ feature^3^ in valence data recorded under similar conditions. P 2s peak areas extracted from Voigt profile fits were used to rescale the signal intensities of the ADP_(aq)_ and ATP_(aq)_ spectra to twice (ADP_(aq)_) and three times (ATP_(aq)_) the intensity of the AMP_(aq)_ spectrum, based on the number of phosphate units in each case. Further details regarding the data treatment are presented in Figure S6 in the SI.

A BE of 195.3 eV (peak 1 in Figure 6) was determined for AMP_(aq)_, corresponding to its single (terminal) phosphate group. Given that the ADP_(aq)_ phosphate chain contains two phosphate units, a second phosphate BE (peak 2) was determined by subtracting the AMP_(aq)_ spectrum from the ADP_(aq)_ data. The difference spectrum is shown using a cyan dashed line. A similar approach was implemented to determine the BE of a third phosphate unit (peak 3) by producing the difference spectrum, ATP_(aq)_ minus ADP_(aq)_, shown as black dashed line in Figure 6. Voigt profile fits to the difference spectra reveal BEs of 196.2 and 196.3 eV for peak 2 and 3, respectively (see Figure S6 in the SI).

As mentioned in the Experiments section, we consider the methodology applied here to provide relative BE values rather than absolute values. Hence, we determined BE energy differences of 900 meV between peaks 1 and 2, and 100 meV between peaks 2 and 3, in agreement with our computations. Based on these results, we assign peak 1 as a PE signature of the terminal phosphate (γ) in ATP_(aq)_, and peaks 2 and 3 as PE signatures of bridging phosphate units (α, β). In doing so, we are assuming that the terminal phosphate P 2s BE value is the same in AMP_(aq)_, ADP_(aq)_, and ATP_(aq)_. The other two phosphate groups, the adenosine–phosphate bridging (α) moiety and the phosphate–phosphate bridging (β) unit, correspond each to different chemical environments, associated with different P 2s BEs.

To assign the BEs extracted from peaks 2 and 3 to such moieties, and to evaluate our assumption more generally, we calculated the P 2s BEs of the individual phosphate units in ADP_(aq)_ ([MgADP]^−^_(aq)_). Our results confirm that the terminal phosphate in ADP_(aq)_ has a lower BE (196.04 eV) compared to the bridging phosphate attached to the nucleoside (196.80 eV). With that in mind, we can assign peaks 2 and 3 as β- and α-phosphate in ATP_(aq)_, respectively. Our calculations also show that our assumption of the BE values of the terminal phosphate units in ADP_(aq)_ and ATP_(aq)_ being of the same magnitude is valid.

In addition, we performed calculations to investigate the effect of Mg^2+^–phosphate interactions on the P core-level BEs of each individual phosphate group in ATP_(aq)_ by calculating the P 2s BEs of α-, β-, and γ- phosphate of [Mg(ATP)_2_]^6–^_(aq)_, [MgATP]^2–^_(aq)_, and Mg_2_ATP_(aq)_ (compare Figure 5(b), 5(c)). For [MgATP]^2–^_(aq)_, we considered two different binding motifs, the cation simultaneously bound to the three phosphate units and the cation bound only to the β- and γ-phosphate groups. The results are summarized in Table 3. Overall, we observe an increase in the P 2s BEs due to Mg^2+^–phosphate interactions, in agreement with the experimental results from the previous section. For all phosphate units in [MgATP]^2–^_(aq)_, changes in the number of phosphate groups interacting with Mg^2+^_(aq)_ lead to BE differences of ∼100 meV between the two binding motif cases. On the other hand, binding to a second Mg^2+^_(aq)_ ion, as in Mg_2_ATP_(aq)_, results in the largest increment in the P 2s BEs, as expected due to the additional positive charge from the metal cation. In addition, the presence of Mg^2+^–phosphate interactions in [MgATP]^2–^_(aq)_ and in Mg_2_ATP_(aq)_ causes the α- and β-phosphate P 2s BEs to adopt different values, as opposed to ATP_(aq)_ in the absence of the divalent cation. Moreover, Table 3 suggests that the energy changes are large enough to be accessed by experiment. But such an attempt would require absolute BEs determination. This will be possible in future studies and opens the possibility of direct determining association equilibrium constants and associated free energies, and thus the effect of divalent cation binding on the ATP hydration free energy, based on careful peak-shape analysis.

### 3.3 ICD Spectroscopy: Intermolecular-Specific Probe of the Mg^2+^_(aq)_ Coordination Environment in the Presence of ATP_(aq)_

The analysis of the Mg^2+^_(aq)_ concentration-dependent valence and core-level spectral changes presented in the previous sections provides site-specific information on the Mg^2+^–ATP_(aq)_ bonding interactions. As explained when introducing Figure 1, ICD spectra, to be presented in the following, are particularly sensitive to interactions between Mg^2+^_(aq)_ and its immediate coordination environment, revealing additional insight into the number and identity of its chelating units.

**Figure 7:**
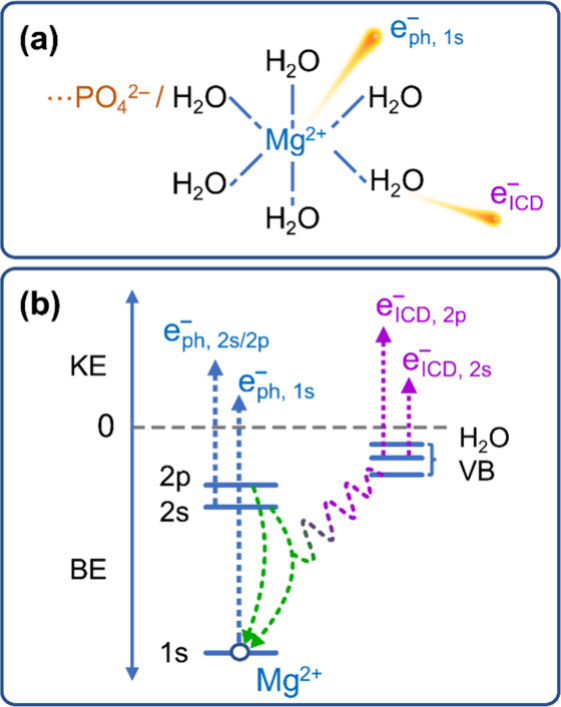
(a) Ejection of the initial 1s photoelectron, 1s e^–^_ph_, from Mg^2+^_(aq)_ and an ICD electron, e^–^_ICD_, associated with the subsequent valence ionization of a hydration-shell water molecule. The sketch also illustrates the direct interaction of Mg^2+^_(aq)_ with a phosphate unit of ATP_(aq)_ which reduces the number of water molecules in the first solvation shell. (b) Energy-level diagram depicting the ICD process studied here. The open circle denotes the 1s core hole upon ionization of Mg^2+^; e^–^_ph_ are the ejected 1s, 2s, and 2p photoelectrons. BE and KE denote electron binding and electron kinetic energies of the measured electrons, respectively. The nonlocal ICD processes, where the relaxation of the metal core hole involves the first solvation shell, lead to the ionization of all water valence (VB) orbitals (and potentially also of phosphate). This produces the ICD electrons e^–^_ICD,2s_ and e^–^_ICD,2p_.

We show here that the ICD signal intensity associated with the hydration shell of a charged atomic ion is proportional to the number of hydrating water molecules and, as a result, the ion–water ICD signal decreases upon replacement of a water molecule by a solute component. In this way, we are sensitive to the quantitative exchange of water molecules by ATP_(aq)_ due to the formation of Mg^2+^–ATP_(aq)_; this is illustrated in Figure 7a, where one water molecule is replaced by one phosphate group in the first solvation shell of Mg^2+^_(aq)_. The associated ICD spectrum further reveals the character of the most involved water orbital,^13^ as we discuss later.

The specific ICD process explored here, and illustrated in Figure 7(b), takes place after the initial photoionization of Mg 1s core-level electrons producing primary photoelectrons e^–^_ph_ (also compare Figure 1). The Mg 1s core hole left behind is refilled by electrons from the Mg 2s (or Mg 2p) core levels, and the released excess energy is used to ionize the surrounding molecules–*i.e.*, the water molecules and the chelating phosphate units in the first coordination shell–producing ICD electrons e^–^_ICD_. (We note that filling the Mg 1s core hole within a local Auger process is more likely, but not of interest here. See ref (13) for a detailed discussion of the core-hole relaxation channels.)

The ICD signal can be well recognized experimentally by constant kinetic energy (KE) of the ICD electrons, *i.e.*, independent of the incident photon energy, just as in the case of Auger electrons. Note further that the ICD signature in the present case occurs in a KE range considerably larger than the respective Auger electrons, which for ICD following Mg^2+^_(aq)_ 1s ionization is near 1190–1250 eV (versus 1160–1190 eV for the respective KLL Auger peak), with ICD processes involving Mg 2p and Mg 2s near 1238 and 1180 eV, respectively.^13^ The much higher kinetic energy of the ICD electrons relative to the respective Auger electrons roughly reflects the considerably larger Mg 2p, 2s BEs than the water valence BEs.

**Figure 8:**
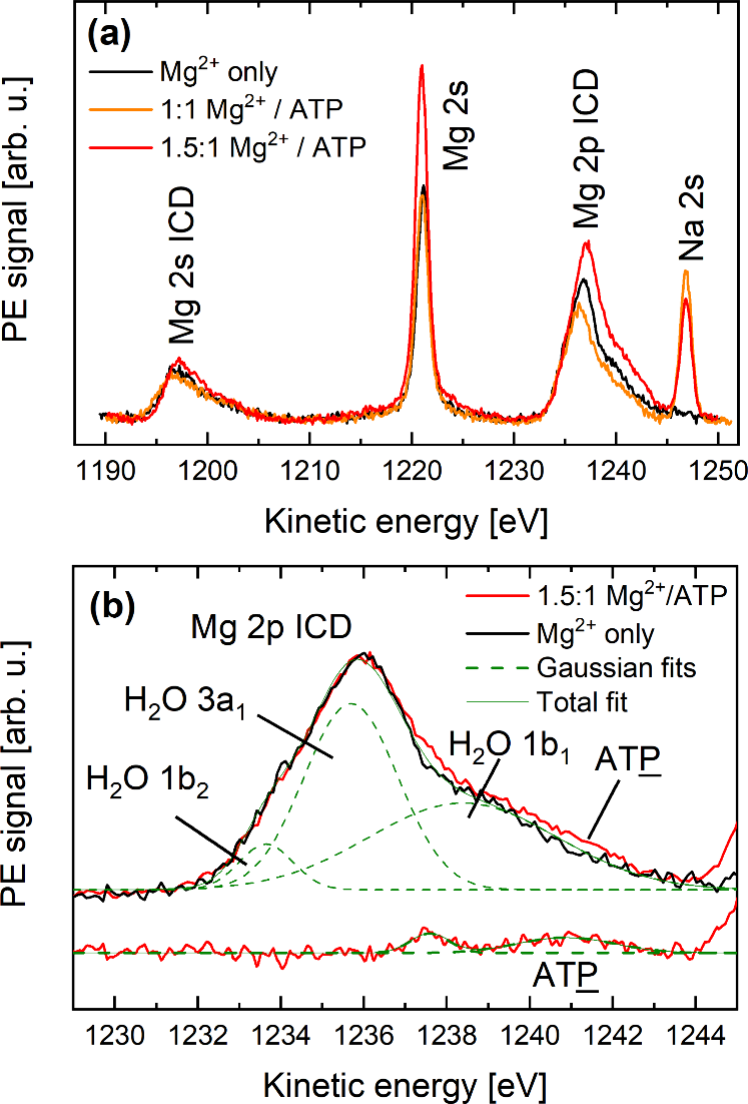
(a) ICD spectra from Mg^2+^_(aq)_ without ATP_(aq)_ and in the presence of ATP_(aq)_ at 1:1 and 1.5:1 Mg^2+^/ATP concentration ratios. The signal intensity has been normalized to the Mg^2+^_(aq)_ concentration (see text). The spectral range of the 2s and 2p ICD channels covers the direct Mg 2s ionization. (b) Close-up of the Mg 2p ICD spectral region presented in panel (a). Spectra in panel (b) are displayed such that the main peaks are at the same KE, and their heights are the same. Gaussian curves (green dashed lines) highlight the individual ionization features from the water orbitals 1b_2_, 3a_1_, and 1b_1_ involved in the ICD process of the Mg^2+^-only data. The Mg^2+^/ATP minus the Mg^2+^-only difference spectrum shown at the bottom (also in red) reveals the ICD signal associated with the Mg^2+^–phosphate interaction (labeled ATP, associated Gaussian fit in green).

Figure 8(a) shows ICD spectra recorded from 0.5 M Mg(NO_3_)_2(aq)_ solutions without ATP_(aq)_ (black line) and with 1:1 and 1.5:1 Mg^2+^/ATP concentration ratios (orange and red lines, respectively). For the 0.5 M Mg(NO_3_)_2(aq)_ solutions (no ATP added), the cation exists in its octahedral configuration ([Mg(H_2_O)_6_]^2+^_(aq)_ (compare Figure 7(a)), regardless of the presence of the counterion (NO_3_^–^_(aq)_).^23,27^ The data were recorded at a photon energy of 1314 eV, which was selected to exceed the Mg 1s BE (based on ref (13)). The secondary-electron background (from inelastically scattered (photo)electrons in solution) was removed by fitting and subsequently subtracting cubic baselines from the different spectral regions. The Mg 2s peak areas extracted from Voigt fits to each data set were used to normalize the signal intensity in all the spectra by scaling the PE signal ordinate based on the Mg^2+^_(aq)_ concentration, assuming equal cross sections. In that way, we normalize the data in Figure 8(a) so as to display the same peak area for the samples where the Mg^2+^_(aq)_ concentration is 0.5 M (black and orange curves) and an area 1.5 times larger for the sample containing 0.75 M Mg^2+^_(aq)_ (red curve). The energy scale in Figure 8(a) was calibrated by first shifting the Mg^2+^_(aq)_-only data to an energy of the main KLL Auger line (not shown in the figure) of 1175.5 eV^13^ (the value for a 2 M aqueous solution of MgCl_2_). The energy scale for the Mg^2+^/ATP_(aq)_ data was then calibrated such that the Mg 2s kinetic energies are consistent with the BEs shown in Figure 5(a). Further details regarding the data treatment can be found in Figure S7 of the SI.

Our first observation is that the Mg 2p and Mg 2s ICD signals occur at slightly but distinctly different KEs in the Mg^2+^/ATP_(aq)_ spectra with respect to the Mg^2+^_(aq)_-only ([Mg(H_2_O)_6_]^2+^_(aq)_) data. This directly reflects the formation of [MgATP]^2–^_(aq)_ (in the 1:1 Mg^2+^/ATP ratio case) and Mg_2_ATP_(aq)_ (in the 1.5:1 Mg^2+^/ATP ratio case) as discussed in connection with Figure 5(b). The observed spectral changes suggest that interactions with ATP_(aq)_ may also affect the transfer of excess energy from Mg^2+^_(aq)_ to its coordinating environment during the ICD process, *e.g.,* due to charge redistribution between the metal ion and the phosphate units in the nucleotide. We note that the Mg^2+^_(aq)_-only sample did not contain Tris_(aq)_, in contrast to the Mg^2+^/ATP solutions, but Mg^2+^–Tris_(aq)_ complexation should be relatively weak, as discussed above.

Our second observation is that the intensity of the Mg 2p ICD feature decreases in the 1:1 Mg^2+^/ATP data compared to the Mg^2+^_(aq)_-only data, despite both of them being associated with equal Mg^2+^_(aq)_ concentrations. This reveals the aforementioned formation of [MgATP]^2–^_(aq)_ and the associated reduction in the number of the Mg^2+^_(aq)_–water ICD channels available. Accordingly, we observe an ∼15% reduction in signal intensity. This corresponds to one water molecule out of six being replaced in [Mg(H_2_O)_6_]^2+^_(aq)_ upon complexation to ATP_(aq)_ (assuming negligible Mg^2+^–Tris_(aq)_ complexation); compare Figure 7(a). The ICD spectral fingerprint associated with the phosphate will be detailed next.

As expected, based on the Mg^2+^_(aq)_ concentration in each sample, the intensity of the Mg 2p ICD signal in the 1.5:1 Mg^2+^/ATP data is ∼1.5 times higher with respect to the 1:1 Mg^2+^/ATP case. However, if we compare the 1.5:1 Mg^2+^/ATP spectra with the Mg^2+^_(aq)_-only data, we observe that the ICD signal intensity in the 1.5:1 Mg^2+^/ATP case is only 1.3 times higher than in the Mg^2+^_(aq)_-only case. This shows that the presence of ATP_(aq)_ causes the signal to decrease with respect to the concentration of the metal cation. In other words, and following our argument from the previous paragraph, we observe an ∼15% reduction in signal due to the formation of [MgATP]^2–^_(aq)_ and Mg_2_ATP_(aq)_.

Another spectral change becomes apparent when overlapping the main peaks (at a common KE and normalized area) of the Mg 2p ICD spectra from both samples, as shown in Figure 8(b) and detailed in the respective figure caption. We recall that the ICD signal is the convolution of ionization peaks associated with all the orbitals involved in the energy transfer process occurring within the coordination shell. The observed broader Mg 2p ICD feature in the Mg^2+^/ATP spectrum with respect to the Mg^2+^_(aq)_-only spectrum thus implies that the ICD process in the latter involves additional orbitals, other than the water orbitals.^13^ In order to validate this conclusion, we subtracted the scaled Mg^2+^_(aq)_-only data from the 1.5:1 Mg^2+^/ATP data. The difference spectrum is shown in red at the bottom of Figure 8(b), highlighting an ICD component associated with ATP (labeled ATP) occurring near 1241 eV KE (see Gaussian fits in green). Note the occurrence of another feature near 1238 eV, the assignment of which is not clear at the moment. Gaussian fits of the individual water orbitals (1b_2_, 3a_1_, and 1b_1_) to the Mg^2+^_(aq)_-only spectrum are shown as dashed lines and are in semiquantitative agreement with ref (13). The ATP (phosphate) contribution from ATP_(aq)_ interacting with Mg^2+^_(aq)_, *i.e.*, due to the formation of species as considered in Figures 5(b), 5(c), is indeed consistent with the lowest phosphate BE being approximately 2 eV lower than that of the water 1b_1_ orbital.^8^ As reported previously, Mg^2+^_(aq)_–water ICD signals show a high specificity for water electrons from the 3a_1_ orbitals.^13^ This is also the case for the data presented here, highlighting the sensitivity of the technique to even orbital spatial orientation.

Overall, the sensitivity of the ICD signal intensity to the charge of the solvated ion involved in the solute–water energy-transfer process allows us to uniquely probe charge-distribution changes upon replacing water molecules in the coordination sphere of [Mg(H_2_O)_6_]^2+^_(aq)_ by phosphate from ATP_(aq)_ to form different Mg^2+^–ATP_(aq)_ complexes.

## 4 Conclusions

We have demonstrated the application of LJ-PES for gaining insight into molecular structure relevant to biophysics and biochemistry. Irradiation of the aqueous-phase sample with soft X-ray photons provides a complex photoemission spectrum resulting from primary and second-order emission photoelectrons that originate from different subcomponents of the molecule and its interaction with its environment. We obtain a comprehensive view on the molecular interactions present in complex solutions even with multicomponent biomolecular systems.

Specifically, we have probed interactions between both the adenine and phosphate units in ATP_(aq)_ with Mg^2+^_(aq)_, as well as the electronic structure of the phosphate chain in ATP_(aq)_, using LJ-PES combined with theoretical calculations, and have correlated our observations to the formation of [MgATP]^2–^_(aq)_, [Mg(ATP)_2_]^6–^_(aq)_, and Mg_2_ATP_(aq)_.

Regarding the photoelectron spectroscopy measurements, valence spectra from solutions with different Mg^2+^/ATP concentration ratios reveal the adenine lowest-ionization peak in ATP_(aq)_ to shift in BE as the Mg^2+^_(aq)_ concentration increases, providing direct evidence of Mg^2+^–adenine and Mg^2+^–phosphate interactions in [MgATP]^2–^_(aq)_, [Mg(ATP)_2_]^6–^_(aq)_, and Mg_2_ATP_(aq)_. A more detailed correlation of spectral energy shifts occurring from the various Mg-ATP_(aq)_ species is revealed from the Mg 2p, Mg 2s, P 2p, and P 2s core-level photoelectron spectra of both the divalent cation and ATP_(aq)_. The interaction between Mg^2+^_(aq)_ cations and the phosphate chain of ATP_(aq)_ is directly reflected in the measured chemical shift in photoelectron spectra for both Mg and P core-level electrons. We also performed a combined analysis of P 2s PE spectra from ATP_(aq)_, ADP_(aq)_, and AMP_(aq)_, and computed BEs to isolate spectral fingerprints of α-, β-, and γ-phosphate in ATP_(aq)_. Our results reveal that the BEs of the bridging groups in the phosphate chain are higher than those of the terminal phosphate. We additionally calculated the P 2s phosphate-specific BEs of [MgATP]^2–^_(aq)_, [Mg(ATP)_2_]^6–^_(aq)_, and Mg_2_ATP_(aq)_, to characterize the effect of different Mg^2+^_(aq)_-binding motifs. These BEs correlate well with the experimental core-level energy shifts.

The ICD study demonstrates the first application of this technique for solving structural problems, with sensitivity and access to interaction details not accessible by photoelectron spectroscopy. In fact, photoelectron spectra are only very weakly dependent on the intermolecular interactions.^86^ This highlights the enormous potential of ICD spectroscopy, and potentially adds an exciting novel tool to probing complex molecular structure in biochemical-relevant aqueous solutions. Since the ICD signal is completely absent for isolated molecules, it inevitably sensitively reports on the intermolecular interactions. Here, we present ICD spectra from ATP_(aq)_ solutions, containing no Mg^2+^_(aq)_ and with a Mg^2+^/ATP ratio of 1.5, allowing us to probe the interactions between the metal cation and a given coordination environment with exceptional sensitivity. Differences in signal intensity and spectral shape for the two solutions identify the replacement of first-hydration-shell water molecules by phosphate from ATP_(aq)_. This suggests a further potential application of ICD-based electron spectroscopy, namely the quantitative determination of solvation-shell constituents.

While we provide a semiquantitative spectral assignment of Mg^2+^–ATP_(aq)_ interactions that shows the ability to distinguish between two speciation models, such information cannot be used, at present, to derive thermodynamic information such as association constants. It would be tempting though, in conjunction with application of more recent methods to determine absolute BEs, to accurately decompose the P 2s peak shape into contributions from the α, β, and γ units, and experimentally quantify the associated energy shifts arising from interaction with Mg^2+^ ions. Furthermore, the combined information extracted from the spectral assignments, chemical shifts, and ICD phenomena provides a foundation for future temperature-dependent PES experiments that could potentially recover the Mg^2+^–phosphate-dependent Mg^2+^–ATP_(aq)_ association equilibria energies and entropy data.

In summary, we have explored the capabilities of state-of-the-art LJ-PES, including direct PE emission and nonlocal relaxation processes upon core-level ionization, in characterizing the electronic-structure interactions between ATP and Mg^2+^ in aqueous solution. Future studies should make use of the structure-sensitivity of ICD to include the respective P- and N-induced relaxation processes. One goal in the context of the present work would be looking for solid experimental evidence of a closed-ring structure, with the adenine–Mg^2+^ interaction potentially showing up in the N 1s ICD spectra.

There are various powerful structural techniques providing analogous structure information, NMR being the prime example. LJ-PES has certain advantages, *e.g*., the surface sensitivity of the technique and capturing the range of instantaneous (rather than time-averaged) structures. However, the full potential of LJ-PES in biophysics still needs to be explored. It is, *e.g.*, imperative to develop empirical guidelines for interpretation of the data without the assistance of *ab initio* theory. It is particularly important to investigate the scope of applicability of the newly emerging ICD spectroscopy, with no previous data to compare. We believe that LJ-PES has a considerable potential to advance complex speciation in biomolecular systems.

## Data Availability

The raw data relevant to this work has been deposited at DOI: 10.5281/zenodo.7998786.

## Supporting Information Available

Additional details regarding sample preparation and predicted speciation, computational methods, treatment, and analysis of the PE spectra and the molecular structures and supporting PE data discussed in the text (PDF)

## ABBREVIATIONS

ADP: adenosine diphosphate
AMP: adenosine monophosphate
ATP: adenosine triphosphate
BE: binding energy
HOMO: highest occupied molecular orbital
HF: Hartree–Fock
ICD: intermolecular Coulombic decay
KE: kinetic energy
LJ-PES: liquid-jet photoelectron/photoemission spectroscopy
MP2: second-order Møller–Plesset
NMR: nuclear magnetic resonance
PCM: polarizable continuum model
PE: photoelectron
PEEK: polyether ether ketone
PES: photoelectron spectroscopy
Tris: (tris(hydroxymethyl)aminomethane)
