# Supplementary material for: How Does Mg2+(aq) Interact with ATP(aq)? Biomolecular Structure through the Lens of Liquid-Jet Photoemission Spectroscopy
Source: J Am Chem Soc. 2024 May 27;146(23):16062–75. doi: 10.1021/jacs.4c03174 (PMC11177255; doi:10.1021/jacs.4c03174)
Supplement: Supplementary file 1 — ja4c03174_si_001.pdf [file ja4c03174_si_001.pdf]

Supporting Information

How Does $Mg^{2+}_{(aq)}$ Interact with $ATP_{(aq)}$? Biomolecular Structure through the Lens of Liquid-Jet Photoemission Spectroscopy

Karen Mudryk,[1] Chin Lee,[1,2,3] Lukáš Tomaník,[4] Sebastian Malerz,[1] Florian Trinter,[1,5] Uwe Hergenhahn,[1] Daniel M. Neumark,[2,3] Petr Slavíček,[4] Stephen Bradforth,[6] and Bernd Winter[1]

[1]Fritz-Haber-Institut der Max-Planck-Gesellschaft, Faradayweg 4-6, 14195 Berlin, Germany

[2]Department of Chemistry, University of California, Berkeley, CA 94720, USA

[3]Chemical Sciences Division, Lawrence Berkeley National Laboratory, Berkeley, CA 94720, USA

[4]Department of Physical Chemistry, University of Chemistry and Technology, Prague, Technická 5, Prague 6 16628, Czech Republic

[5]Institut für Kernphysik, Goethe-Universität Frankfurt, Max-von-Laue-Straße 1, 60438 Frankfurt am Main, Germany

[6]Department of Chemistry, University of Southern California, Los Angeles, CA 90089, USA

Table of contents

Sample preparation 2

Possible ‘open form’ and ‘closed form’ configuration of $Mg_2ATP_{(aq)}$ 2

Predicted composition of the $ATP_{(aq)}$ samples with dissolved Mg^{2+} as a function of the Mg^{2+}/ATP concentration ratio 2

Photoelectron spectra from $Tris_{(aq)}$ solutions with and without dissolved Mg^{2+} 5

Valence photoelectron spectra of $ATP_{(aq)}$, $ADP_{(aq)}$ and $AMP_{(aq)}$ 6

Valence photoelectron spectra of $ADP_{(aq)}$ with added Mg^{2+} 6

Linear baselines to the P 2s data presented in Figure 4e in the main text 7

Data treatment to determine α-, β-, and γ-phosphate P 2s binding energies in $ATP_{(aq.)}$ 8

Intermolecular Coulombic decay (ICD) spectra of $ATP_{(aq)}$ solutions with dissolved Mg^{2+} 9

Sample input for calculations of P 2s binding energies using Q-Chem, version 6.0 10

Cartesian coordinates of the structures used to calculate P 2s, Mg 2s, Mg 2p, and valence binding energies 14

References 31

Sample preparation

Table S1. $Mg(NO_3)_{2(aq)}$ and $Tris_{(aq)}$ (tris(hydroxymethyl)aminomethane) concentrations in the $ATP_{(aq)}$, $ADP_{(aq)}$, and $AMP_{(aq)}$ solutions used in this work. The solution pH was 8.2 in all samples. All values are in mol dm^{-3}.

Mg^{2+} / ATP ratio	$Mg(NO_3)_{2(aq)}$	$Tris_{(aq)}$		
		0.5 M $ATP_{(aq)}$	0.5 M $ADP_{(aq)}$	0.5 M $AMP_{(aq)}$
0	0	1.16	1.36	0.23
0.25	0.125	1.21	1.52	
0.5	0.25	1.32	1.70	
0.75	0.375	1.43	1.77	
1	0.5	1.54	1.93	
1.5	0.75	1.65	2.34	

Possible 'open form' and 'closed form' configuration of $Mg_2ATP_{(aq)}$

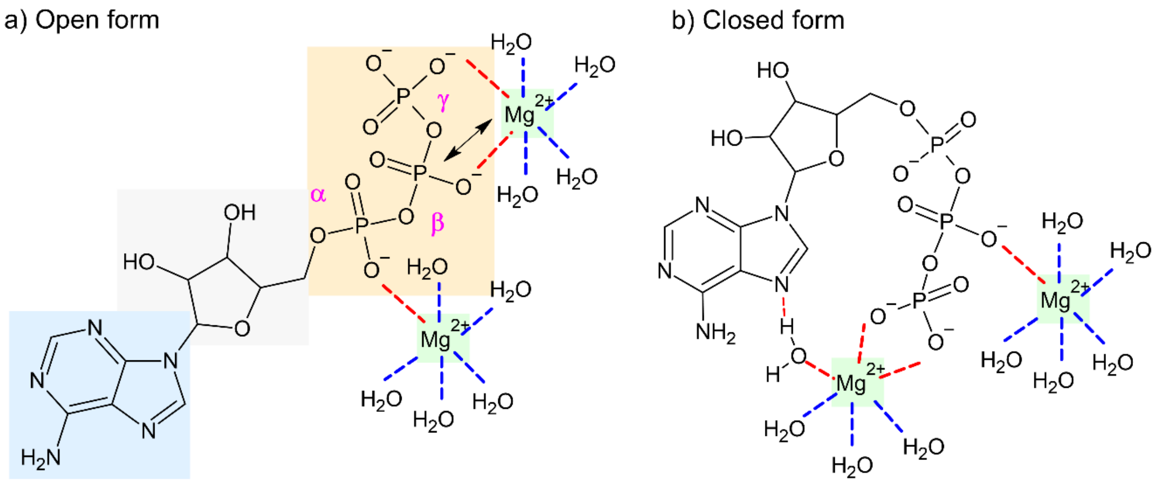

Figure S1. Possible molecular structures adopted by $Mg_2ATP_{(aq.)}$ a) Open form. b) Closed form. This figure was produced using the ACD / ChemSketch software [1].

Predicted composition of the $ATP_{(aq)}$ samples with dissolved Mg^{2+} as a function of the Mg^{2+}/ATP concentration ratio

The composition of the $ATP_{(aq)}$ samples with dissolved Mg^{2+} was calculated as follows. First, we assumed the species in solution to be ATP^{4-}, $HATP^{3-}$, H_2ATP^{2-}, $MgATP^{2-}$, Mg_2ATP, $MgHATP^{-}$, MgH_2ATP, Mg^{2+}, Na^{+}, H^{+} and OH^{-}, with a solution pH of 8.2 to match the experimental conditions. Thus,

$$[H^+] = 10^{-8.2} \quad (1)$$

We used equilibrium constants from Storer and Cornish-Bowden [2] – namely, Equations (1), (2), (4), (5), (6) and (7) from Table 2 in Storer's paper. Notably, the values correspond to solutions of lower ionic strength (0.1 M) than those studied here. Nevertheless, they still serve to allow a qualitative estimate for our system.

$$\frac{[H_2ATP^{2-}]}{[H^+]\cdot[HATP^{3-}]} = 8.5\cdot 10^3 \quad (2)$$

$$\frac{[HATP^{3-}]}{[H^+]\cdot[ATP^{4-}]} = 1.09\cdot 10^7 \quad (3)$$

$$\frac{[MgH_2ATP]}{[Mg^{2+}]\cdot[H_2ATP^{2-}]} = 2.0\cdot 10^1 \quad (4)$$

$$\frac{[MgHATP^{-}]}{[Mg^{2+}]\cdot[HATP^{3-}]} = 5.42\cdot 10^2 \quad (5)$$

$$\frac{[MgATP^{2-}]}{[Mg^{2+}]\cdot[ATP^{4-}]} = 3.48\cdot 10^4 \quad (6)$$

$$\frac{[Mg_2ATP]}{[Mg^{2+}]\cdot[MgATP^{2-}]} = 4.0\cdot 10^1 \quad (7)$$

In addition, we used equations for mass balance, where $c_0(Na_2H_2ATP)$ is the initial concentration of ATP compound and $c_0(Mg(NO_3)_2)$ is the initial concentration of Mg salt.

$$c_0(Na_2H_2ATP) = [ATP^{4-}] + [HATP^{3-}] + [H_2ATP^{2-}] + [MgATP^{2-}] + [Mg_2ATP] + [MgHATP^{-}] + [MgH_2ATP] \quad (8)$$

$$c_0(Mg(NO_3)_2) = [Mg^{2+}] + [MgATP^{2-}] + 2\cdot[Mg_2ATP] + [MgHATP^{-}] + [MgH_2ATP] \quad (9)$$

$$[Na^+] = 2\cdot c_0(Na_2H_2ATP) \quad (10)$$

Finally, we considered the self-ionization of water at 25 °C

$$[H^+]\cdot[OH^-] = 10^{-14} \quad (11)$$

This represents a system of 11 equations with 11 unknowns, which was solved for different Mg^{2+}/ATP concentration ratios using the Maple 2016 software [3]. The results are reported in Table S2.

The extended speciation plot was created considering the additional complex $Mg(ATP)_2^{6-}$ and the respective equilibrium constant

$$\frac{[MgATP^{2-}]\cdot[ATP^{4-}]}{[Mg(ATP)_2{}^{6-}]} = 6.2\cdot 10^{-3} \quad (12)$$

taken from Bock et al.[4] The system of 12 equations with 12 unknowns was solved and the results are reported in Table S3.

Table S2. Calculated concentration of species as a function of Mg^{2+}/ATP concentration ratio at a solution pH of 8.2. The values were determined as described in the text, using equilibrium constants from Reference [2]. The total concentration of $ATP_{(aq)}$ was kept at 0.5 mol dm^{-3} while the total concentration of $Mg^{2+}_{(aq)}$ was varied in the range 0.125–0.75 mol dm^{-3}. All values are in mol dm^{-3}.

	**0.25:1 ratio**	**0.5:1 ratio**	**0.75:1 ratio**	**1:1 ratio**	**1.5:1 ratio**
$\mathbf{ATP^{4-}_{(aq)}}$	3.51E-01	2.34E-01	1.18E-01	1.57E-02	3.66E-04
$\mathbf{H^{+}_{(aq)}}$	6.30957E-09	6.30957E-09	6.30957E-09	6.30957E-09	6.30957E-09
$\mathbf{[H_2ATP]^{2-}_{(aq)}}$	1.29E-06	8.64E-07	4.36E-07	5.80E-08	1.35E-09
$\mathbf{[HATP]^{3-}_{(aq)}}$	2.41E-02	1.61E-02	8.14E-03	1.08E-03	2.52E-05
$\mathbf{Mg^{2+}_{(aq)}}$	1.02E-05	3.06E-05	9.03E-05	8.54E-04	2.12E-02
$\mathbf{Mg_2ATP_{(aq)}}$	5.10E-05	3.04E-04	1.34E-03	1.59E-02	2.29E-01
$\mathbf{[MgATP]^{2-}_{(aq)}}$	0.1247542	0.249093647	0.371824746	0.466768697	0.270137983
$\mathbf{MgH_2ATP_{(aq)}}$	2.64455E-10	5.2803E-10	7.88196E-10	9.89459E-10	5.7264E-10
$\mathbf{[MgHATP]^{-}_{(aq)}}$	1.34E-04	2.67E-04	3.98E-04	5.00E-04	2.89E-04
$\mathbf{OH^{-}_{(aq)}}$	1.58E-06	1.58E-06	1.58E-06	1.58E-06	1.58E-06

Table S3. Calculated concentration of species (including $Mg(ATP)_2^{6-}$) as a function of Mg^{2+}/ATP concentration ratio at a solution pH of 8.2. The values were determined as described in the text, using equilibrium constants from References [2] and [4]. The total concentration of $ATP_{(aq.)}$ was kept at 0.5 mol dm^{-3} while the total concentration of $Mg^{2+}_{(aq)}$ was varied in the range 0.125–0.75 mol dm^{-3}. All values are in mol dm^{-3}.

	**0.25:1 ratio**	**0.5:1 ratio**	**0.75:1 ratio**	**1:1 ratio**	**1.5:1 ratio**
$\mathbf{ATP^{4-}_{(aq)}}$	0.2344094666	0.014865245	0.00099912	0.000509229	0.000165
$\mathbf{H^{+}_{(aq)}}$	6.30957E-09	6.30957E-09	6.30957E-09	6.30957E-09	6.30957E-09
$\mathbf{[H_2ATP]^{2-}_{(aq)}}$	8.65E-7	5.48E-8	3.69E-9	1.88E-9	6.09E-10
$\mathbf{[HATP]^{3-}_{(aq)}}$	0.01612136	0.00102235	0.000068714	0.00003502	0.00001135
$\mathbf{Mg^{2+}_{(aq)}}$	6.51E-8	0.0000304504	0.00451273783	0.01138740914	0.03288926626
$\mathbf{Mg_2ATP_{(aq)}}$	1.38E-9	0.0000191866	0.02832293427	0.09191835433	0.2484479051
$\mathbf{[MgATP]^{2-}_{(aq)}}$	0.000530987	0.01575231338	0.1569054937	0.2017982168	0.1888518150
$\mathbf{MgH_2ATP_{(aq)}}$	1.13E-12	3.34E-11	3.33E-10	4.28E-10	4.00E-10
$\mathbf{[MgHATP]_{(aq)}}$	5.69E-7	0.0000168729	0.0001680679	0.000216154	0.000202287
$\mathbf{OH^{-}_{(aq)}}$	1.58E-06	1.58E-06	1.58E-06	1.58E-06	1.58E-06
$\mathbf{[Mg(ATP)_2]^{6-}_{(aq)}}$	0.1244683764	0.2341619901	0.1567678318	0.1027615107	0.03116082140

Photoelectron spectra from $Tris_{(aq)}$ solutions with and without dissolved Mg^{2+}

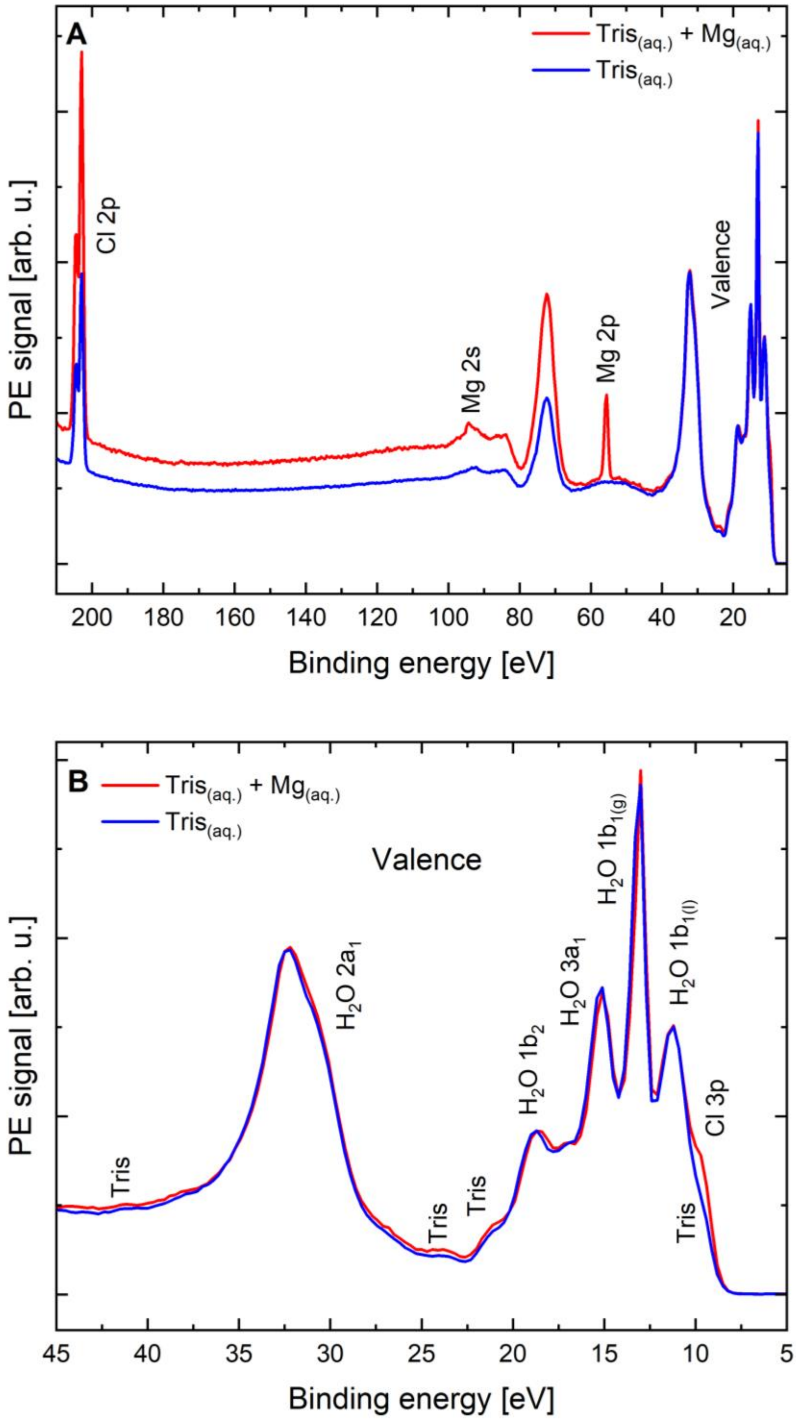

Figure S2. a) Photoelectron spectra recorded from 3.2 M $Tris_{(aq)}$ without $Mg^{2+}_{(aq)}$ (blue line) and with 0.5 M $MgCl_{2(aq)}$ (red line) using a photon energy of 250 eV. The sample pH was adjusted to 8.2 by addition of concentrated HCl in both cases. The binding energy (BE) scale was calibrated based on the liquid water solvent valence $1b_1$ peak position, and spectral intensities are displayed to yield its same height. b) Highlight of the valence spectral region from panel a. Photoelectron signatures of water and $Cl^{-}_{(aq)}$ are labelled according to References [5] and [6], respectively.

Valence photoelectron spectra of $ATP_{(aq)}$, $ADP_{(aq)}$ and $AMP_{(aq)}$

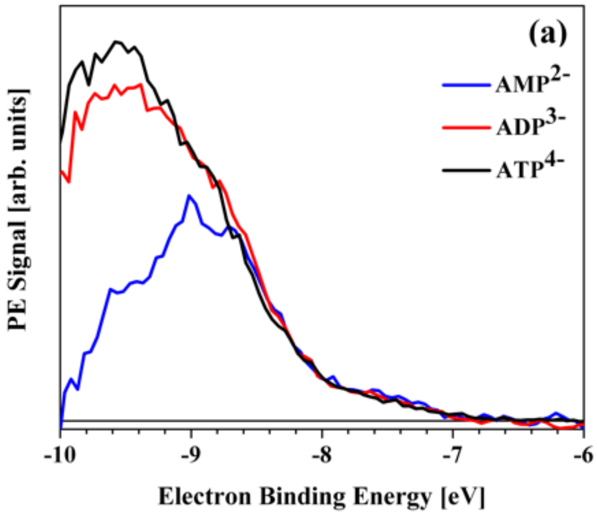

Figure S3. Valence photoelectron signals from $AMP_{(aq)}$, $ADP_{(aq)}$, and $ATP_{(aq)}$ solutions without disolved Mg^{2+} isolated by subtracting the solvent water contributions, facilitating the identification of the phosphate feature and its changes in signal intensity across the nucleotide series. The figure was extracted from Reference [7]. The experimental conditions used are described in Reference [8].

Valence photoelectron spectra of $ADP_{(aq)}$ with added Mg^{2+}

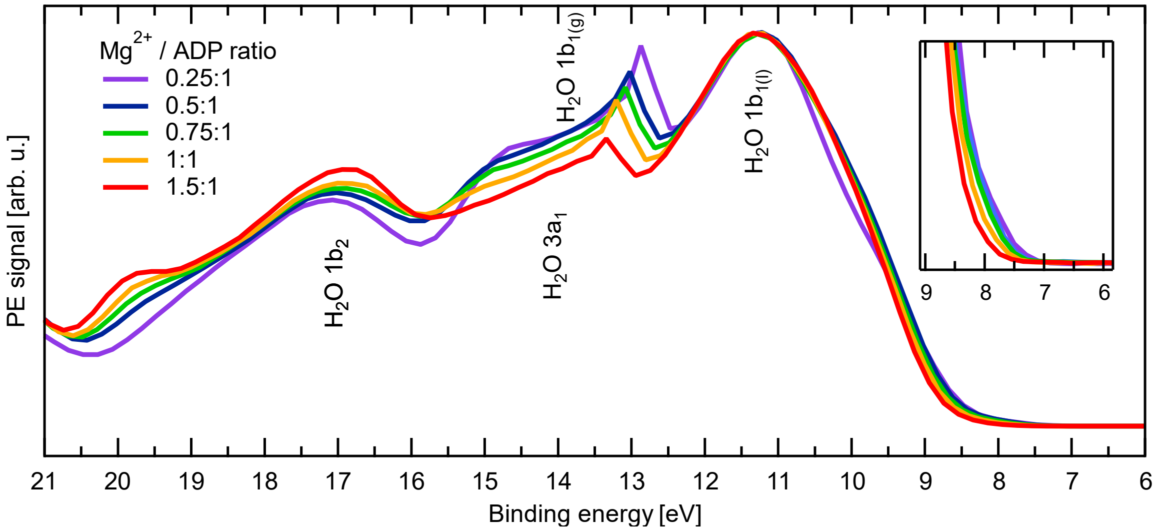

Figure S4. Valence photoelectron spectra from 0.5 M $ADP_{(aq)}$ samples containing $Mg^{2+}{}_{(aq)}$ as a function of the Mg^{2+}/ADP concentration ratio recorded using a photon energy of 250 eV. The sample pH was 8.2, adjusted by addition of Tris. The BE scale was calibrated based on the liquid water solvent valence $1b_1$ peak position, and spectral intensities are displayed to yield its same height. The figure inset highlights chemical shifts for the adenine unit, hinting to the presence of phosphate–Mg^{2+}–adenine interactions, as reported in Reference [9]; signal intensity is scaled analogous to the data shown in Figure 4(a) of the main manuscript.

Linear baselines to the P 2s data presented in Figure 4e in the main text

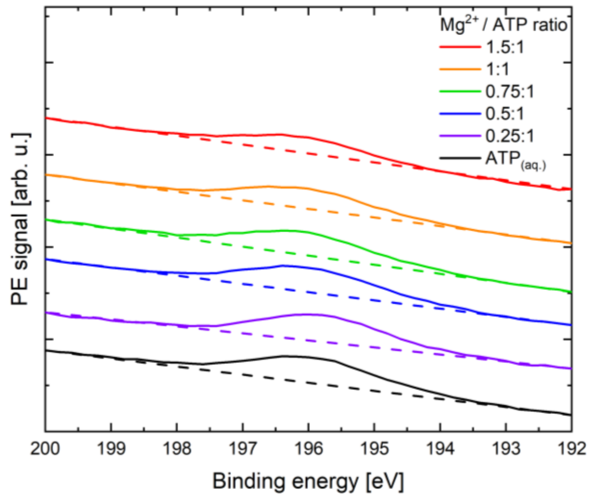

Figure S5. Linear baselines subtracted to the data presented in Figure 4e in the main text. The data are shown with a vertical offset for a better comparison.

Data treatment to determine α-, β-, and γ-phosphate P 2s binding energies in $ATP_{(aq)}$

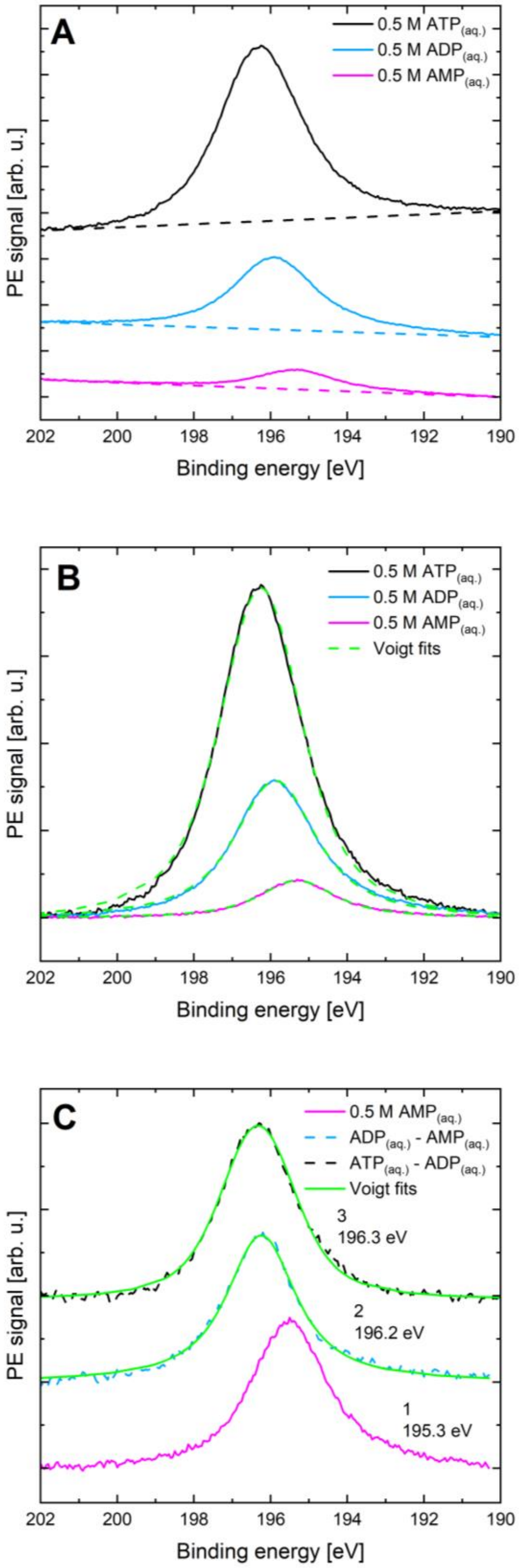

Figure S6. a) Linear baselines fit to and subtracted from the P 2s $AMP_{(aq)}$, $ADP_{(aq)}$ and $ATP_{(aq)}$ spectra. b) Voigt fits to the background-subtracted data performed to extract peak areas to re-scale each spectrum based on the number of phosphate units in each sample, as explained in the main text. c) Voigt fits to the $AMP_{(aq.)}$ and the $ADP_{(aq)}-AMP_{(aq)}$ and $ATP_{(aq)}-ADP_{(aq)}$ difference spectra. The data are shown with a vertical offset for a better comparison. Peaks 1, 2 and 3 correspond to γ-, β-, and α-phosphate, as described in the main text.

Intermolecular Coulombic decay (ICD) spectra of $ATP_{(aq)}$ solutions with dissolved Mg^{2+}

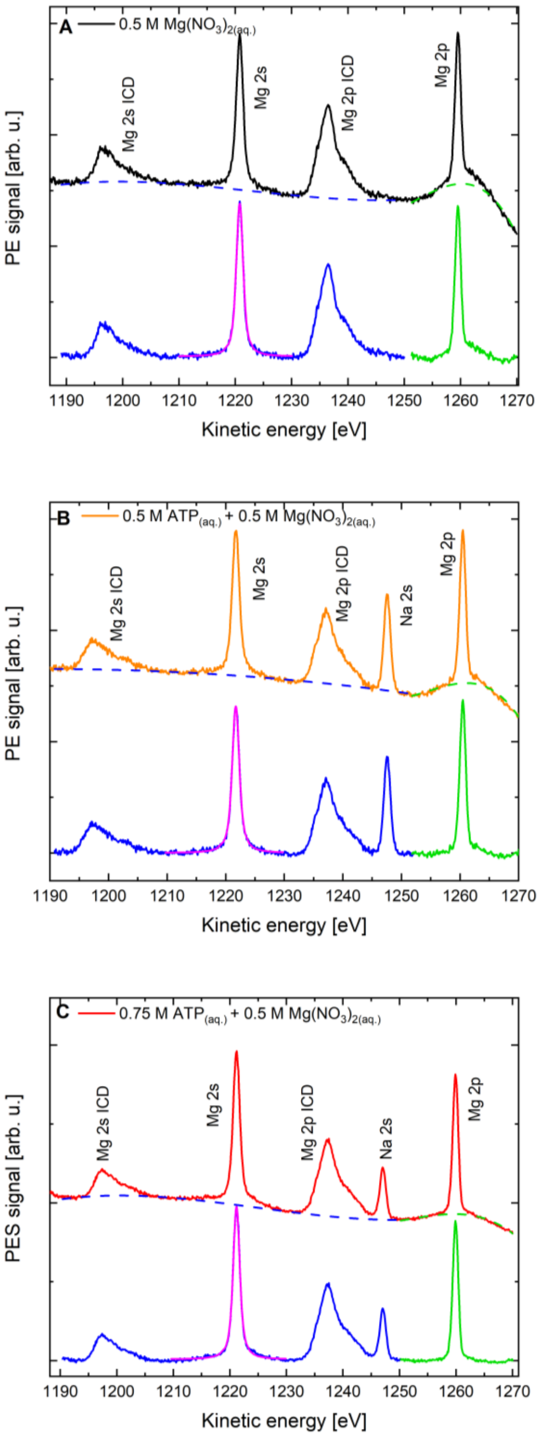

Figure S7. ICD spectra from a 0.5 M $Mg(NO_3)_{2(aq)}$ solution (panel a) and samples additionally containing $ATP_{(aq)}$ at 1:1 and 1.5:1 Mg^{2+}/ATP concentration ratios (panels b and c, respectively). Cubic baselines were fit to and subtracted from different spectral regions – highlighted in green and blue dashed lines – to obtain the background-free data shown at the bottom of each plot and in Figure 8 in the main text (the use of a single baseline per spectrum did not allow the background to be removed completely, particularly in the vicinity of the Mg 2p peak). Voigt profile fits to the Mg 2s feature, used to extract peak areas to normalize the signal intensity according to the $Mg^{2+}_{(aq)}$ concentration, are shown in magenta. Kinetic energy is shown without the corrections delineated in the main article.

Sample input for calculations of P 2s binding energies using Q-Chem, version 6.0

```
$molecule
-2 1
 C    7.224927   -0.822676    1.178578
 C    6.400689    0.390773    1.648327
 O    5.741657    0.889906    0.512408
 C    6.357295    0.400165   -0.665258
 C    6.764706   -1.015146   -0.271981
 N    5.429865    0.126595    2.680502
 C    4.426729   -0.802826    2.709145
 C    3.760179   -0.587106    3.893105
 N    4.331220    0.463841    4.589294
 C    5.297970    0.850033    3.838275
 N    4.132054   -1.736044    1.805532
 C    3.098102   -2.463627    2.163904
 N    2.364894   -2.366194    3.267622
 C    2.673168   -1.429240    4.163609
 N    1.957320   -1.353158    5.298273
 C    5.383016    0.475700   -1.817277
 O    5.034723    1.825785   -2.085047
 P    5.533519    2.671333   -3.330859
 O    7.086128    2.959546   -2.998166
 P    7.796586    4.068300   -2.077932
 O    9.114600    3.311294   -1.667383
 P   10.678216    3.215316   -2.178902
 O   10.922827    4.458632   -3.017769
 O    7.777934   -1.506934   -1.105013
 O    8.578676   -0.448645    1.231810
 O    4.774182    3.950285   -3.325847
 O    5.528548    1.824879   -4.556795
 O    8.111996    5.247946   -2.943544
 O    6.986746    4.312065   -0.857579
 O   11.454269    3.203851   -0.883496
 O   10.774878    1.951695   -2.989088
 O    7.892952    3.910242    1.866903
 O    8.886263    6.585860    1.873713
 O    4.444713    3.630963    0.526843
 O    6.631657    7.046811   -0.114316
 O    2.995563    0.236236   -4.751444
 O    7.395943   -1.171897   -7.894599
 O    7.824774    0.477340   -5.580742
 O    4.831772    2.698903   -7.194269
 O    2.431757    3.795051   -1.615895
 O    4.636346    6.771471   -2.335407
 O    1.385435    1.259172   -2.503023
 O    4.035588    5.163317   -5.855433
 O   12.966150    4.476538   -5.001197
 O   14.234390    4.236107   -1.054033
 O   13.102625    0.908295   -0.257338
 O   10.128784    2.142256    1.496512
 O   11.223501    5.922243    0.264295
 O   13.307154    0.620968   -3.152611
 O    9.350816   -0.462957   -3.353000
 O   10.034819    2.394841   -5.715182
 O   15.023858    2.932689   -3.590595
 O   10.321438    7.235626   -2.025491
 O    6.733315    6.081292   -5.374094
 O    9.472906    5.150173   -5.608173
Mg    9.851872    6.021003   -3.696028
```

H	*2.801346*	*-3.237732*	*1.480314*
H	*2.035870*	*-0.538121*	*5.866455*
H	*1.088072*	*-1.840026*	*5.337770*
H	*5.967154*	*1.657172*	*4.057903*
H	*7.068832*	*1.137048*	*2.046875*
H	*7.057422*	*-1.696537*	*1.791669*
H	*9.083442*	*-1.045094*	*0.688867*
H	*5.903130*	*-1.666458*	*-0.293672*
H	*7.705345*	*-2.449706*	*-1.196279*
H	*7.241875*	*0.986102*	*-0.886453*
H	*5.815846*	*0.010135*	*-2.691753*
H	*4.465763*	*-0.036247*	*-1.564339*
H	*0.468714*	*1.380529*	*-2.720858*
H	*3.103148*	*3.902189*	*-2.288935*
H	*4.740231*	*5.856743*	*-2.593883*
H	*4.110315*	*4.771779*	*-4.985102*
H	*3.820253*	*0.716911*	*-4.739126*
H	*5.122023*	*2.366727*	*-6.342873*
H	*7.053876*	*0.926309*	*-5.228815*
H	*6.666984*	*-1.751152*	*-7.707936*
H	*6.785323*	*6.124739*	*-0.322195*
H	*5.173605*	*3.989064*	*0.023919*
H	*7.563655*	*3.969013*	*0.970381*
H	*8.535770*	*5.702781*	*2.004410*
H	*13.327405*	*3.942895*	*-0.983119*
H	*11.243794*	*4.997076*	*0.018199*
H	*10.514336*	*2.459124*	*0.678530*
H	*12.526669*	*1.657321*	*-0.424827*
H	*14.536007*	*2.115559*	*-3.482834*
H	*12.451211*	*1.046945*	*-3.232718*
H	*9.814979*	*0.346478*	*-3.128514*
H	*10.344013*	*2.228645*	*-4.821276*
H	*10.657318*	*6.801758*	*-1.232143*
H	*12.299761*	*4.271847*	*-4.340567*
H	*6.796653*	*5.660194*	*-4.519793*
H	*8.570916*	*5.300176*	*-5.884832*
H	*2.481603*	*0.582441*	*-4.023542*
H	*7.518432*	*-0.640673*	*-7.106659*
H	*5.614821*	*2.787914*	*-7.724618*
H	*8.259211*	*0.066565*	*-4.830605*
H	*4.197890*	*4.439183*	*-6.458247*
H	*4.757724*	*7.277308*	*-3.130167*
H	*2.937496*	*3.704587*	*-0.808888*
H	*1.692518*	*2.102843*	*-2.164560*
H	*4.659113*	*2.704974*	*0.598189*
H	*8.656785*	*3.335200*	*1.816173*
H	*8.258972*	*7.017199*	*1.299901*
H	*5.957097*	*7.303932*	*-0.737174*
H	*10.476687*	*6.050143*	*0.849382*
H	*9.690837*	*1.319313*	*1.296189*
H	*12.578179*	*0.273310*	*0.215853*
H	*14.571194*	*3.829778*	*-1.850518*
H	*13.410962*	*0.503367*	*-2.210164*
H	*14.479139*	*3.473358*	*-4.159442*
H	*8.845222*	*-0.714022*	*-2.584581*
H	*9.265208*	*1.836569*	*-5.818751*
H	*9.666441*	*7.863729*	*-1.741839*
H	*12.703440*	*4.018541*	*-5.791878*
H	*5.841635*	*5.917281*	*-5.682034*
H	*9.630392*	*4.202328*	*-5.678875*

```
 O    11.583857     6.941532   -4.587323
 H    12.055758     7.491578   -3.970534
 H    12.216189     6.299676    -4.913845
 O     8.753769     7.733178    -4.382556
 H     9.228149     8.347153    -4.933370
 H     7.950080     7.504803    -4.851660
$end

$rem
METHOD HF
BASIS General
THRESH 12
MAX_SCF_CYCLES 129
solvent_method  pcm
PCM_PRINT      1
MEM_TOTAL  384000
MEM_STATIC 2000
$end

$basis
H 0
aug-cc-pVTZ
****
C 0
aug-cc-pVTZ
****
O 0
aug-cc-pVTZ
****
N 0
aug-cc-pVTZ
****
P 0
aug-cc-pCVTZ
****
Mg 0
aug-cc-pVTZ
****
$end

$pcm
THEORY            IEFPCM
RADII             Bondi
vdwScale          1.2
NonEquilibrium
$end

$solvent
Dielectric        78.39
OpticalDielectric 1.78
$end

@@@
$molecule
-1 2
READ
$end
$rem
METHOD HF
BASIS General
```

```
THRESH 12
MAX_SCF_CYCLES 129
unrestricted TRUE
mom_start   1
MOM_METHOD IMOM
scf_guess read
solvent_method  pcm
PCM_PRINT      1
MEM_TOTAL  384000
MEM_STATIC 2000
$end

$basis
H 0
aug-cc-pVTZ
****
C 0
aug-cc-pVTZ
****
O 0
aug-cc-pVTZ
****
N 0
aug-cc-pVTZ
****
P 0
aug-cc-pCVTZ
****
Mg 0
aug-cc-pVTZ
****
$end

$pcm
THEORY           IEFPCM
RADII            Bondi
vdwScale         1.2
StateSpecific     Marcus
$end

$solvent
Dielectric       78.39
OpticalDielectric 1.78
$end

$occupied
1:265
1:58 60:265
$end
```

Cartesian coordinates of the structures used to calculate P 2s, Mg 2s, Mg 2p, and valence binding energies

ATP^{4-}

O	2.475207	-3.395307	2.768565
C	3.116643	-2.171512	3.011723
C	4.194717	-2.008883	1.924691
C	4.177935	-3.368016	1.207509
C	2.757811	-3.863434	1.459706
N	3.592196	-2.147772	4.372745
C	4.552282	-2.919846	4.966112
C	4.579243	-2.523986	6.283538
N	3.647530	-1.526251	6.511357
C	3.094416	-1.345473	5.366960
N	5.321334	-3.857766	4.415860
C	6.144257	-4.397138	5.286787
N	6.275861	-4.116745	6.578892
C	5.501338	-3.174173	7.114557
N	5.660240	-2.861670	8.412372
O	3.762556	-0.965352	1.096142
O	4.441559	-3.287136	-0.169428
C	2.609052	-5.367908	1.432164
O	1.257592	-5.732666	1.663907
P	0.321919	-6.381603	0.554373
O	-1.008187	-6.594551	1.188953
O	0.993617	-7.558026	-0.068742
O	0.260261	-5.166480	-0.489728
P	-0.375405	-4.956620	-1.955765
O	-1.253088	-6.112303	-2.277146
O	-0.980812	-3.593681	-1.953029
O	0.956906	-4.993922	-2.814477
P	1.431486	-4.531127	-4.329516
O	1.507941	-3.022919	-4.302705
O	2.798210	-5.183288	-4.453890
O	0.402891	-5.085745	-5.285297
O	-1.950813	-3.896050	-6.208541
O	3.369355	-1.764794	-2.602713
O	-4.815969	-7.715183	-0.363696
O	1.171972	-6.227337	-7.798200
O	-0.066080	-0.677557	-4.447338
O	-0.329941	-9.806943	-1.223062
O	-2.513122	-2.186627	-3.927615
O	3.926770	-5.658181	-7.051304
O	3.362062	-8.234585	-1.563689
O	4.442487	-5.642485	-2.161818
O	-1.076998	-6.252734	4.049765
O	1.166574	-7.970530	4.602655
O	3.029755	-1.813375	-6.445368
O	-3.698626	-5.550410	1.249475
O	-3.269544	-3.086843	-0.123536
O	-1.664018	-1.818526	1.950515
O	0.009658	-1.377793	-0.344203
O	-0.290018	-3.447608	3.925356
O	1.419191	0.382854	-2.192692
O	2.864183	-8.078403	-4.448286
O	1.626454	-9.012748	-6.928543

O	-2.451152	-9.059588	0.636424
O	1.975078	-9.354465	2.127703
O	5.013537	-3.153315	-4.641851
H	6.796334	-5.165080	4.913079
H	4.953714	-2.328459	8.870541
H	6.179552	-3.492127	8.984392
H	2.313712	-0.641374	5.161558
H	2.412108	-1.358978	2.924072
H	5.164218	-1.778983	2.344231
H	4.335341	-0.895551	0.341894
H	4.888333	-4.029205	1.684220
H	5.379139	-3.215837	-0.314038
H	2.076008	-3.407579	0.752399
H	2.952773	-5.750967	0.482470
H	3.198696	-5.813628	2.220668
H	-1.118061	-6.430238	3.109629
H	0.592011	-3.503467	3.566691
H	-2.789173	-5.849289	1.250759
H	-1.983174	-8.265615	0.896974
H	0.912742	-8.581517	5.284363
H	0.156337	-9.044528	-0.902593
H	2.563180	-8.034850	-1.076572
H	1.681047	-8.824445	1.390148
H	-2.558492	-3.269788	-0.736625
H	-2.306234	-2.255737	1.391350
H	-0.250710	-2.101641	-0.914611
H	-2.058230	-2.681111	-3.244459
H	2.427856	-2.180773	-5.796466
H	0.443952	-1.491402	-4.447315
H	0.872055	0.172392	-2.950488
H	2.701292	-2.239892	-3.109062
H	3.582167	-5.471233	-6.174368
H	4.356278	-3.847058	-4.599979
H	3.853624	-5.447558	-2.894940
H	2.809894	-7.121409	-4.477703
H	-1.130256	-4.294734	-5.897970
H	0.850963	-5.829439	-6.985799
H	-4.600024	-7.330510	-1.213263
H	2.024280	-8.829576	-6.079103
H	0.418860	-7.381479	4.481141
H	1.696649	-8.887439	2.913933
H	-0.512242	-9.635718	-2.139404
H	3.813274	-7.394948	-1.651559
H	-1.890087	-9.460721	-0.025083
H	-4.181022	-6.211003	0.753530
H	-0.795029	-2.946504	3.283477
H	-0.841854	-5.326339	4.107245
H	-0.995615	-1.524300	1.333096
H	0.467357	-0.747213	-0.897957
H	-2.431064	-2.722281	-4.716296
H	-3.493661	-3.928017	0.276237
H	-0.960771	-0.960535	-4.265626
H	2.180614	-0.189848	-2.274852
H	3.508452	-2.259853	-1.800260
H	3.885953	-2.095804	-6.137286
H	4.766434	-2.560258	-3.936695

H	4.327454	-4.852197	-7.354662
H	4.331957	-4.934790	-1.534834
H	2.952167	-8.297053	-3.521721
H	-1.722774	-3.391847	-6.980429
H	2.106160	-6.027763	-7.802235
H	-4.075683	-8.275210	-0.128717
H	1.397894	-8.155122	-7.283145
H	-3.085445	-6.169534	-2.604806
H	-0.462198	-6.833069	-5.070542
O	-0.959272	-7.570695	-4.717995
H	-1.139700	-7.282010	-3.825103
O	-4.028635	-6.285738	-2.748432
H	-4.116345	-6.689773	-3.603566

$[Mg(ATP)_2]^{6-}$

C	15.122721	5.138014	-3.504118
N	13.906459	4.566719	-3.274397
C	12.978463	5.501577	-3.646856
N	13.492853	6.597815	-4.084327
C	14.857403	6.392645	-4.002849
C	13.695646	3.225794	-2.745279
C	13.655959	3.154269	-1.208974
C	12.152796	3.108730	-0.923158
C	11.660327	2.306619	-2.111870
O	12.463419	2.756673	-3.192572
C	10.236685	2.530503	-2.511529
O	9.447062	1.864144	-1.564477
P	8.058127	2.398362	-1.017189
O	8.143991	3.826765	-0.643504
O	11.889457	2.575883	0.357547
O	14.223319	1.953109	-0.763739
O	7.629475	1.416794	0.008996
Mg	7.277821	-0.619513	0.235947
O	6.542116	-0.761625	-1.652732
P	5.526419	-0.766091	-2.760918
O	6.008052	-1.474981	-4.011919
O	7.011058	-2.731423	0.507198
P	6.142188	-3.593151	1.357725
O	6.182023	-5.060980	1.166976
O	4.650139	-3.038669	1.205605
C	3.473581	-3.815468	1.414581
C	3.243752	-4.160418	2.868156
C	2.066049	-5.097165	3.119522
C	1.738992	-4.831982	4.604703
C	2.335936	-3.422040	4.835981
O	2.891889	-3.009358	3.612834
N	1.414841	-2.415669	5.295979
C	0.254853	-1.980160	4.713765
C	-0.177077	-0.933963	5.495298
N	0.699507	-0.717399	6.545268
C	1.611613	-1.606421	6.381914
N	-0.354311	-2.447602	3.625852
C	-1.456730	-1.785844	3.353718
N	-1.987882	-0.763065	4.014993
C	-1.367394	-0.307689	5.102385

N	-1.923525	0.701920	5.794503
O	2.280263	-5.763823	5.489029
O	2.423516	-6.413040	2.818043
O	6.383011	-3.241808	2.909289
P	7.634561	-3.192847	3.911696
O	7.272420	-3.979698	5.131771
O	8.071605	-0.535251	2.135405
P	7.693606	-0.227748	3.556581
O	6.277872	0.307161	3.696041
O	8.718909	0.561546	4.332315
O	7.667652	-1.678156	4.351118
O	8.880105	-3.591552	3.196954
O	7.163961	2.277224	-2.346436
P	5.576165	2.216176	-2.621152
O	4.823489	2.344435	-1.354096
O	5.407556	0.798225	-3.287956
O	5.288968	3.209570	-3.708924
O	4.129284	-1.143834	-2.343945
O	13.453017	2.315472	2.808501
O	9.425963	-0.296207	6.922565
O	3.337847	-0.060487	3.553376
O	10.796150	-2.290349	1.542425
O	11.269579	-4.628879	0.020866
O	11.602513	0.765424	4.373071
O	5.873058	3.150462	3.397641
O	7.671667	-5.599681	-3.347842
O	5.195813	0.461980	6.374736
O	2.695263	1.811472	7.068843
O	12.700419	-1.806246	3.665144
O	7.032244	-0.355753	8.525155
O	8.904493	-3.686264	-1.477933
O	11.222240	-3.885209	5.018588
O	9.380493	-3.226530	7.173552
O	9.911080	-6.053320	2.210080
O	5.585024	-2.522216	7.076878
O	8.708855	3.396166	3.886662
O	7.958028	-7.184363	0.333688
O	5.233082	-7.296355	2.738408
O	5.171714	-6.058666	5.365088
O	8.777708	-6.637859	5.073210
H	-2.002917	-2.107345	2.486061
H	-1.368880	1.190363	6.463363
H	-2.644196	1.229116	5.350426
H	2.466447	-1.735190	7.013407
H	3.109003	-3.488982	5.586823
H	0.675322	-4.838949	4.778696
H	3.231766	-5.832860	5.401951
H	1.222619	-4.786651	2.514473
H	1.671923	-6.988752	2.896904
H	4.140100	-4.595735	3.289555
H	3.543228	-4.726858	0.839419
H	2.658389	-3.215312	1.039463
H	6.894418	-5.151112	-3.680245
H	8.239205	-3.558572	-0.800139
H	7.309273	-6.492084	0.443350
H	5.599118	-6.589954	2.205269

H	10.521191	-3.883869	4.368628
H	9.489560	-5.233948	2.474692
H	11.291042	-3.802824	0.510617
H	10.088979	-2.629774	2.096079
H	8.640149	-3.508179	6.639479
H	6.118903	-2.981935	6.429409
H	5.806243	-5.343355	5.334948
H	8.323192	-5.798207	5.096767
H	10.652971	0.678913	4.267172
H	8.808623	2.453470	4.046224
H	7.880934	-0.286171	8.089511
H	9.180961	-0.005652	6.036329
H	4.267426	0.040041	3.744427
H	2.007647	1.166513	6.933982
H	5.655510	0.485307	5.533309
H	5.941906	2.195097	3.439275
H	13.096563	2.389663	1.924657
H	12.207221	-1.902899	2.851275
H	8.079500	-4.993869	-2.731702
H	4.287771	-7.190708	2.668681
H	9.650418	-4.098528	-1.044069
H	8.643849	-6.953484	0.956939
H	10.378742	-1.658413	0.961722
H	12.108829	-4.710725	-0.417305
H	10.537239	-5.797659	1.535887
H	10.777370	-3.679279	5.839241
H	9.390331	-2.271661	7.118297
H	5.250605	-1.746240	6.636125
H	5.313004	-6.554580	4.559313
H	9.249930	-6.650342	4.247052
H	7.778891	3.499760	3.689979
H	6.694974	-1.216141	8.284260
H	10.236271	0.151458	7.136227
H	11.970934	-0.102727	4.202663
H	5.862767	0.455737	7.058054
H	3.509382	1.383351	6.806049
H	3.194736	-0.999391	3.474705
H	5.492138	3.362202	2.547132
H	12.783967	1.859401	3.320709
H	12.317237	-2.456972	4.252614
H	10.059966	2.101246	-3.487768
H	9.993399	3.583286	-2.540051
H	11.759206	4.117314	-0.998480
H	14.145408	4.004835	-0.754462
H	14.495834	2.614531	-3.132882
H	11.838995	1.250632	-1.945347
H	11.168941	3.089791	0.785453
H	15.170804	2.009521	-0.795295
H	11.931646	5.306381	-3.580631
N	16.331728	4.614211	-3.298705
C	17.292568	5.443459	-3.639176
N	17.183460	6.672802	-4.131665
C	15.967360	7.183678	-4.326565
H	18.297853	5.088404	-3.505079
N	15.858576	8.441332	-4.788328
H	16.669169	8.867457	-5.182710

H	14.978680	8.755342	-5.135152
O	9.279387	-0.967748	-0.578293
H	9.296387	-1.710180	-1.181603
H	9.541796	-0.192303	-1.069819
H	2.325789	3.294184	-6.149083
H	0.885710	0.625091	-1.431151
H	3.268509	-2.648227	-2.847018
H	5.651851	-3.256993	-4.332366
H	5.130943	-1.069390	-5.575432
H	7.629637	-0.934966	-4.711083
H	1.772295	-4.619872	-2.016357
H	5.078375	5.113043	-3.161387
H	6.873340	4.031126	-4.569015
H	3.359321	3.259062	-3.968764
H	3.473369	1.179684	0.593260
H	4.657122	3.392850	0.148916
H	9.244449	4.239346	0.805459
H	6.921926	5.244031	-0.359312
O	5.359108	-0.231104	1.070584
H	4.817547	-1.016001	1.101030
H	5.542164	0.006090	1.984167
H	9.287225	6.308592	-3.018087
H	10.155320	3.242373	-8.631937
H	5.198593	2.339493	-5.534371
H	7.126298	1.847552	-6.663620
H	13.890636	4.036335	3.510037
H	2.660434	-0.075290	-2.883271
O	14.157138	4.892007	3.850850
H	13.356159	5.390936	3.956311
O	9.943975	4.296103	1.444816
H	9.583821	3.970587	2.271228
O	6.257400	5.921835	-0.219878
H	5.577839	5.496061	0.297064
O	4.436953	3.901070	0.933899
H	3.618606	3.517986	1.236609
O	1.238452	-5.260748	-1.547845
H	1.012676	-4.855234	-0.719768
O	9.416528	6.289534	-3.958971
H	8.831081	5.611751	-4.292506
O	9.639252	2.486624	-8.885695
H	9.119353	2.253960	-8.115737
O	8.077183	1.832393	-6.550585
H	8.264880	1.031211	-6.057745
O	8.464824	-0.643217	-5.094734
H	9.071753	-0.565787	-4.368010
O	5.010833	6.023032	-2.877133
H	5.415714	6.051349	-2.011373
O	7.626788	4.338730	-5.067684
H	7.966797	3.560476	-5.504564
O	2.421581	3.130841	-4.123140
H	1.972521	3.762477	-3.572943
O	2.719646	1.401988	1.131653
H	2.858149	0.920103	1.948224
O	0.448099	0.757347	-0.589911
H	1.146564	0.954536	0.032205
O	5.400157	-4.179386	-4.439086

H	4.539110	-4.231532	-4.031733
O	4.714992	-0.801526	-6.402789
H	3.802607	-1.057091	-6.335677
O	2.827070	-3.451517	-3.154714
H	2.291041	-3.185862	-3.893235
O	5.191203	2.025902	-6.438664
H	4.966458	1.094410	-6.396698
O	1.846360	0.381491	-3.112698
H	2.093385	1.248825	-3.426677
O	2.581254	3.242391	-7.067314
H	3.453200	2.852624	-7.042568

$[Mg\text{-}ATP]^{2-}$ (Mg^{2+} bound to α-,β-, and γ-phosphate)

C	0.954635	-3.123957	3.443092
C	1.610854	-1.889433	3.533673
C	2.789449	-1.766177	2.834651
N	3.356160	-2.711252	2.086101
C	2.654092	-3.822032	2.074538
N	1.508199	-4.078531	2.695855
N	1.315758	-0.714236	4.201936
C	2.287604	0.072025	3.904475
N	3.229610	-0.495530	3.083724
C	4.391895	0.185018	2.568644
O	4.242835	0.366270	1.182247
C	5.473575	0.158313	0.513960
C	6.155354	-0.917085	1.351917
C	5.737815	-0.535146	2.777581
O	6.619309	0.403858	3.330351
O	7.543763	-0.887231	1.156396
C	5.220621	-0.256326	-0.917086
O	4.847572	0.858966	-1.721345
P	5.789484	1.577778	-2.784268
O	6.984715	0.754435	-3.087353
N	-0.184353	-3.394308	4.104090
O	4.870864	1.946999	-3.908973
O	6.278113	2.897527	-2.038129
P	5.823876	4.448014	-1.974217
O	6.846183	5.111237	-1.112622
O	5.644011	4.967985	-3.356215
O	4.471795	4.388940	-1.167305
P	2.848690	4.363266	-1.480705
O	2.273622	3.883838	-0.170176
O	2.473143	5.777729	-1.840163
O	2.660675	3.386401	-2.615988
O	4.226696	4.755847	-5.816853
O	6.428076	4.179170	-7.531633
O	5.797239	7.788283	-3.992978
O	9.037713	0.648255	-0.981255
O	5.813876	-1.040892	-5.171126
O	5.609770	1.480859	-6.719840
O	9.141228	2.015070	-4.648081
O	9.046779	3.412046	0.024391
O	9.244509	6.024718	-2.708953
O	6.329650	8.091998	-1.109363
O	6.278299	5.336834	1.747569

O	2.947777	1.272365	-7.804559
O	3.140877	-0.194173	-4.501277
O	7.966584	4.676847	-5.088271
Mg	2.867314	1.752391	-3.688918
H	3.051403	-4.630130	1.488144
H	-0.714854	-2.639517	4.481100
H	-0.697209	-4.207287	3.839005
H	2.393032	1.084118	4.242168
H	4.427132	1.141974	3.064286
H	5.644278	-1.392764	3.429316
H	7.512979	0.142518	3.136512
H	5.755873	-1.890504	1.106083
H	7.915831	-1.752578	1.281506
H	6.063134	1.067817	0.540104
H	6.104656	-0.719004	-1.327820
H	4.401396	-0.958707	-0.960809
H	6.342901	-0.625570	-4.491640
H	5.404007	1.741385	-5.822270
H	8.465148	1.553130	-4.156892
H	8.420425	0.650216	-1.711753
H	5.773822	6.843921	-3.838579
H	7.174615	4.740860	-4.553539
H	5.625908	4.489096	-7.106086
H	4.628638	4.723901	-4.946163
H	6.574670	7.170756	-1.046940
H	6.465825	5.258845	0.812505
H	8.320828	3.889258	-0.370453
H	8.508768	5.763186	-2.159212
H	1.707619	6.362084	-3.443250
H	0.730087	6.483577	-1.214766
H	3.865125	7.650362	1.361416
H	3.124921	7.258201	-0.976322
H	3.871636	1.252867	-7.546872
H	4.009729	-0.547629	-4.711939
H	1.825137	2.132131	0.000714
H	3.342771	3.390641	5.372239
H	3.065684	4.255242	1.474931
H	0.630110	4.277967	0.628412
H	-0.686783	4.208028	-5.020045
H	3.757463	6.661855	-6.012344
H	5.931473	-0.487630	-5.938829
H	8.613946	0.147937	-0.288697
H	6.010330	2.247528	-7.125850
H	8.803038	2.900373	-4.771067
H	2.622088	-0.902624	-4.136851
H	2.692880	0.368674	-7.950677
H	3.547236	4.088126	-5.810015
H	6.464123	4.590023	-8.387786
H	7.656990	4.587318	-5.987826
H	5.989805	8.155332	-3.132621
H	5.407970	8.126914	-0.856794
H	5.511906	4.790034	1.902835
H	9.016749	2.527743	-0.336788
H	9.045734	5.676645	-3.573110
O	-0.110281	6.781431	-0.867759
H	-0.332542	6.139911	-0.196873

O	4.153383	7.361749	2.226547
H	5.013855	6.971798	2.087350
O	3.482357	8.026677	-0.515964
H	2.942277	8.768045	-0.763970
O	1.272554	6.691656	-4.233959
H	1.965647	7.068193	-4.774832
O	3.463097	4.497936	2.316446
H	3.425215	5.451950	2.355871
O	2.579862	3.373276	4.807144
H	2.868759	3.734246	3.967829
O	1.598700	1.196017	0.029647
H	2.375341	0.766625	0.377667
O	-0.205282	4.547645	1.019815
H	-0.825788	3.856791	0.819887
O	0.106611	4.426524	-5.495140
H	0.468923	5.208172	-5.067267
O	3.542788	7.578891	-5.845920
H	4.210357	7.871481	-5.227066
O	2.129293	2.516227	-5.431968
H	2.226428	2.064690	-6.276298
H	1.378198	3.119680	-5.480290
O	1.302677	0.762907	-2.684577
H	0.413279	0.975813	-2.949080
H	1.338145	0.870184	-1.723505

$[Mg\text{-}ATP]^{2-}$ (Mg^{2+} bound to β- and γ-phosphate)

C	7.224927	-0.822676	1.178578
C	6.400689	0.390773	1.648327
O	5.741657	0.889906	0.512408
C	6.357295	0.400165	-0.665258
C	6.764706	-1.015146	-0.271981
N	5.429865	0.126595	2.680502
C	4.426729	-0.802826	2.709145
C	3.760179	-0.587106	3.893105
N	4.331220	0.463841	4.589294
C	5.297970	0.850033	3.838275
N	4.132054	-1.736044	1.805532
C	3.098102	-2.463627	2.163904
N	2.364894	-2.366194	3.267622
C	2.673168	-1.429240	4.163609
N	1.957320	-1.353158	5.298273
C	5.383016	0.475700	-1.817277
O	5.034723	1.825785	-2.085047
P	5.533519	2.671333	-3.330859
O	7.086128	2.959546	-2.998166
P	7.796586	4.068300	-2.077932
O	9.114600	3.311294	-1.667383
P	10.678216	3.215316	-2.178902
O	10.922827	4.458632	-3.017769
O	7.777934	-1.506934	-1.105013
O	8.578676	-0.448645	1.231810
O	4.774182	3.950285	-3.325847
O	5.528548	1.824879	-4.556795
O	8.111996	5.247946	-2.943544
O	6.986746	4.312065	-0.857579

O	11.454269	3.203851	-0.883496
O	10.774878	1.951695	-2.989088
O	7.892952	3.910242	1.866903
O	8.886263	6.585860	1.873713
O	4.444713	3.630963	0.526843
O	6.631657	7.046811	-0.114316
O	2.995563	0.236236	-4.751444
O	7.395943	-1.171897	-7.894599
O	7.824774	0.477340	-5.580742
O	4.831772	2.698903	-7.194269
O	2.431757	3.795051	-1.615895
O	4.636346	6.771471	-2.335407
O	1.385435	1.259172	-2.503023
O	4.035588	5.163317	-5.855433
O	12.966150	4.476538	-5.001197
O	14.234390	4.236107	-1.054033
O	13.102625	0.908295	-0.257338
O	10.128784	2.142256	1.496512
O	11.223501	5.922243	0.264295
O	13.307154	0.620968	-3.152611
O	9.350816	-0.462957	-3.353000
O	10.034819	2.394841	-5.715182
O	15.023858	2.932689	-3.590595
O	10.321438	7.235626	-2.025491
O	6.733315	6.081292	-5.374094
O	9.472906	5.150173	-5.608173
Mg	9.851872	6.021003	-3.696028
H	2.801346	-3.237732	1.480314
H	2.035870	-0.538121	5.866455
H	1.088072	-1.840026	5.337770
H	5.967154	1.657172	4.057903
H	7.068832	1.137048	2.046875
H	7.057422	-1.696537	1.791669
H	9.083442	-1.045094	0.688867
H	5.903130	-1.666458	-0.293672
H	7.705345	-2.449706	-1.196279
H	7.241875	0.986102	-0.886453
H	5.815846	0.010135	-2.691753
H	4.465763	-0.036247	-1.564339
H	0.468714	1.380529	-2.720858
H	3.103148	3.902189	-2.288935
H	4.740231	5.856743	-2.593883
H	4.110315	4.771779	-4.985102
H	3.820253	0.716911	-4.739126
H	5.122023	2.366727	-6.342873
H	7.053876	0.926309	-5.228815
H	6.666984	-1.751152	-7.707936
H	6.785323	6.124739	-0.322195
H	5.173605	3.989064	0.023919
H	7.563655	3.969013	0.970381
H	8.535770	5.702781	2.004410
H	13.327405	3.942895	-0.983119
H	11.243794	4.997076	0.018199
H	10.514336	2.459124	0.678530
H	12.526669	1.657321	-0.424827
H	14.536007	2.115559	-3.482834

H	12.451211	1.046945	-3.232718
H	9.814979	0.346478	-3.128514
H	10.344013	2.228645	-4.821276
H	10.657318	6.801758	-1.232143
H	12.299761	4.271847	-4.340567
H	6.796653	5.660194	-4.519793
H	8.570916	5.300176	-5.884832
H	2.481603	0.582441	-4.023542
H	7.518432	-0.640673	-7.106659
H	5.614821	2.787914	-7.724618
H	8.259211	0.066565	-4.830605
H	4.197890	4.439183	-6.458247
H	4.757724	7.277308	-3.130167
H	2.937496	3.704587	-0.808888
H	1.692518	2.102843	-2.164560
H	4.659113	2.704974	0.598189
H	8.656785	3.335200	1.816173
H	8.258972	7.017199	1.299901
H	5.957097	7.303932	-0.737174
H	10.476687	6.050143	0.849382
H	9.690837	1.319313	1.296189
H	12.578179	0.273310	0.215853
H	14.571194	3.829778	-1.850518
H	13.410962	0.503367	-2.210164
H	14.479139	3.473358	-4.159442
H	8.845222	-0.714022	-2.584581
H	9.265208	1.836569	-5.818751
H	9.666441	7.863729	-1.741839
H	12.703440	4.018541	-5.791878
H	5.841635	5.917281	-5.682034
H	9.630392	4.202328	-5.678875
O	11.583857	6.941532	-4.587323
H	12.055758	7.491578	-3.970534
H	12.216189	6.299676	-4.913845
O	8.753769	7.733178	-4.382556
H	9.228149	8.347153	-4.933370
H	7.950080	7.504803	-4.851660

Mg_2ATP

C	4.804353	-2.044697	2.701936
C	3.876445	-0.891776	2.280688
O	4.622018	-0.061753	1.423674
C	6.001809	-0.379617	1.484242
C	6.021542	-1.875544	1.783454
N	2.652832	-1.277726	1.621047
C	2.474243	-2.055614	0.510115
C	1.117379	-2.065474	0.282912
N	0.458978	-1.302476	1.231278
C	1.398245	-0.861098	1.986650
N	3.392489	-2.690404	-0.216019
C	2.860173	-3.355109	-1.216816
N	1.578346	-3.451237	-1.552825
C	0.670651	-2.813736	-0.814591
N	-0.629742	-2.942833	-1.127521
C	6.675508	-0.022183	0.180986

O	6.796909	1.399933	0.080616
P	6.235297	2.312314	-1.077266
O	7.571068	2.721022	-1.885626
P	8.433327	4.029580	-2.127009
O	9.887806	3.521138	-1.875696
P	11.321475	3.653127	-2.723855
O	11.112631	4.887803	-3.595123
O	7.223982	-2.236468	2.400910
O	5.150003	-1.820654	4.041949
O	5.709644	3.555181	-0.415827
O	5.342659	1.580368	-1.995899
O	8.246764	4.470205	-3.546439
O	8.077043	5.063826	-1.093594
O	12.356882	3.840869	-1.661488
O	11.433976	2.378364	-3.517332
O	9.713503	5.174158	1.404418
O	12.230864	7.350195	-2.251523
O	15.112046	3.708783	-2.466214
O	13.328584	1.659443	-0.057317
O	12.470492	5.863210	0.323821
O	5.642065	-1.003469	-3.450953
O	8.413788	-0.724556	-4.227877
O	2.624781	2.740919	-1.830359
O	5.062460	2.206651	-4.935351
O	3.340549	5.402558	-2.623285
O	3.268873	2.671399	1.078026
O	14.045374	0.981916	-2.870891
O	10.375003	-0.069331	-2.231987
O	13.088559	2.674845	-5.957276
O	9.619907	1.628424	-5.636770
O	8.557662	8.028516	-2.204608
O	5.459659	5.086721	-4.657410
O	9.533903	4.335150	-6.458750
O	9.249968	7.291647	-4.765676
O	11.976710	4.533614	-7.783921
Mg	9.587825	5.319249	-4.716460
Mg	6.399835	5.338064	-0.096349
O	5.042777	6.616770	-0.780055
O	7.032802	5.851096	1.734625
H	3.543754	-3.891299	-1.849023
H	-1.289395	-2.313371	-0.725203
H	-0.861864	-3.318085	-2.021692
H	1.259320	-0.225029	2.837334
H	3.582662	-0.341848	3.161135
H	4.329922	-3.010147	2.592997
H	5.990478	-2.232022	4.213933
H	5.869249	-2.439883	0.874818
H	7.465607	-3.122978	2.162373
H	6.466972	0.161944	2.300684
H	7.675730	-0.428620	0.164149
H	6.112873	-0.405042	-0.654313
H	14.211303	3.891632	-2.192413
H	12.313867	7.044711	-1.351113
H	12.467881	5.120780	-0.283507
H	12.965685	2.442298	-0.474499
H	10.213543	1.828022	-4.907365

H	12.564175	2.599979	-5.159284
H	13.178979	1.312016	-3.101922
H	10.732207	0.736986	-2.601656
H	3.437697	2.277367	-2.025101
H	5.170884	1.976561	-4.016429
H	8.620939	0.023945	-4.782392
H	5.471818	-0.206892	-2.955973
H	4.028637	3.174520	0.798353
H	3.038883	4.528262	-2.358127
H	9.487713	5.192485	0.477869
H	8.529655	7.184312	-1.763699
H	8.955037	7.691732	-3.933781
H	12.446954	3.895501	-7.239905
H	6.307144	5.015089	-4.227297
H	9.440902	3.384160	-6.328654
H	6.552660	-0.929552	-3.735458
H	8.989790	-0.628177	-3.469743
H	2.572828	2.726203	-0.876651
H	5.162073	3.156136	-4.966635
H	10.462553	0.009721	-1.282196
H	14.604890	1.755583	-2.881667
H	10.141847	1.149180	-6.271569
H	13.988965	2.777371	-5.671468
H	11.684828	5.755331	0.852784
H	11.896260	6.599747	-2.737010
H	13.694439	1.167224	-0.788328
H	15.306488	4.320078	-3.166784
H	8.822555	7.747197	-5.484681
H	12.066454	4.244227	-8.685332
H	9.683682	4.246291	1.639857
H	7.708403	8.437288	-2.042735
H	3.606477	1.790550	1.217718
H	2.575596	5.867931	-2.943690
H	4.816272	5.247538	-3.969252
H	10.296580	4.452803	-7.038427
H	5.273276	7.512535	-1.046483
H	4.390073	6.276122	-1.404727
H	6.696727	6.567479	2.265073
H	7.978734	5.761306	1.899928
O	5.930027	9.173328	-1.580384
O	12.771620	7.289343	-7.199866
H	13.467870	7.260605	-6.554727
H	12.555961	6.378697	-7.393429
H	5.959206	9.830981	-0.892722
H	5.463091	9.575138	-2.306195
H	8.347178	2.152908	1.353137
O	9.155302	2.323084	1.830587
H	8.973963	2.096378	2.736471
H	10.342063	0.926161	1.027361
O	10.902707	0.275293	0.610465
H	11.746330	0.708590	0.473458

Mg-ADP⁻

C	-5.727742	-2.073887	1.617346
C	-5.224173	-0.847915	2.073160
C	-3.876909	-0.631798	1.896894
N	-3.016721	-1.479160	1.334261
C	-3.598978	-2.591996	0.947199
N	-4.878976	-2.931823	1.052421
N	-5.814779	0.239375	2.692804
C	-4.846308	1.062750	2.872635
N	-3.633790	0.606724	2.423419
C	-2.398543	1.350543	2.474325
O	-2.010089	1.691152	1.168649
C	-0.609985	1.548361	0.998441
C	-0.243573	0.406305	1.939616
C	-1.206082	0.618738	3.116842
O	-0.664460	1.490885	4.070920
O	1.105361	0.481168	2.315691
C	-0.317268	1.278175	-0.457548
O	-0.732403	2.412986	-1.194019
P	-0.792454	2.451343	-2.787461
O	0.721488	2.585087	-3.244411
P	1.815139	3.827202	-3.145187
O	2.562101	3.618876	-1.859478
N	-7.015103	-2.431326	1.759827
O	-1.305224	1.160342	-3.319535
O	-1.550783	3.695474	-3.123086
Mg	-0.932277	5.604549	-3.398920
O	-0.834704	5.411240	-5.522421
O	2.652811	3.639048	-4.392242
O	0.986312	5.093620	-3.176283
O	-2.935261	6.331387	-3.758045
O	-1.333555	6.005480	-1.337051
O	2.974333	1.116462	-5.571482
O	1.822089	5.287023	-6.505766
O	-3.082198	4.305478	0.130031
O	-1.575569	0.602299	-6.088784
O	2.262469	4.856680	0.669446
O	5.387176	3.420986	-1.693213
O	0.886826	1.083143	-7.554150
O	1.365529	3.609508	-8.804159
O	2.937550	2.761254	2.641401
O	1.830780	7.957636	-5.380714
O	5.410157	4.185569	-4.553409
H	-2.964359	-3.324531	0.483245
H	-7.690875	-1.732284	1.978716
H	-7.342371	-3.220232	1.245308
H	-4.932558	2.026979	3.330946
H	-2.607391	2.243619	3.042705
H	-1.501858	-0.310888	3.582916
H	0.225162	1.220208	4.269619
H	-0.452987	-0.543973	1.471042
H	1.466462	-0.387893	2.444381
H	-0.112602	2.459946	1.310587
H	0.745258	1.116502	-0.590825
H	-0.858241	0.404355	-0.792564
H	0.063304	0.948062	-7.084748

H	1.579346	1.039109	-6.893777
H	3.848566	1.100179	-5.942343
H	2.878904	1.974455	-5.143496
H	-3.226296	6.434420	-4.664989
H	-3.643079	5.904199	-3.287566
H	-1.489096	6.894436	-1.038894
H	-1.924410	5.432999	-0.839757
H	-1.485736	0.792121	-5.150847
H	-1.834734	-0.309723	-6.148185
H	0.034192	5.413381	-5.930931
H	-1.443654	5.856499	-6.107496
H	2.147652	4.696589	-5.816533
H	1.759576	4.768175	-7.308811
H	2.661166	8.121305	-4.948353
H	1.925309	7.115143	-5.825646
H	5.787328	3.545866	-5.145463
H	4.463635	4.010942	-4.537550
H	5.628073	3.657913	-2.585416
H	4.427518	3.463099	-1.688997
H	2.329955	2.029760	2.551140
H	2.812203	3.096705	3.521531
H	2.072884	3.469590	-9.422863
H	1.168509	2.749229	-8.425246
H	2.483685	4.169770	1.294233
H	-3.437200	4.662849	0.935819
H	-2.696640	3.463287	0.363982
H	2.326216	4.455964	-0.200859
O	-0.329561	7.655164	-3.551831
H	0.414535	7.800801	-4.144194
H	-0.995957	8.289667	-3.792203
H	-2.879153	0.258163	-2.774953
O	-3.674661	-0.220093	-2.532703
H	-3.615523	-0.361552	-1.595523
O	-3.203709	6.676603	-6.587334
H	-3.800074	6.159670	-7.119010
H	-3.249495	7.563521	-6.929016

ATP^{4-} (for valence-ionization calculations)

C	-2.400558	0.703779	5.179742
C	-1.639049	1.418460	4.047763
O	-1.537477	0.507256	2.965714
C	-1.850156	-0.815965	3.408783
C	-2.873870	-0.608153	4.523536
N	-2.254726	2.638068	3.567285
C	-3.551119	2.834866	3.142775
C	-3.606353	4.157934	2.719384
N	-2.368910	4.762856	2.866975
C	-1.604144	3.827684	3.364102
N	-4.562770	1.960829	3.129693
C	-5.677445	2.495980	2.644561
N	-5.868479	3.746405	2.202622
C	-4.844856	4.611427	2.231082
N	-5.063939	5.898288	1.837632
C	-2.372804	-1.627764	2.252020
O	-1.303659	-1.870314	1.338756
P	-1.603465	-2.638537	-0.065133

O	-0.257493	-2.755909	-0.745376
O	-2.833756	-1.695303	5.430226
O	-1.483809	0.426406	6.222334
O	-2.144292	-4.097608	0.429502
P	-1.488680	-5.492236	1.012285
O	-0.808102	-6.216751	-0.138368
O	-2.753772	-1.997422	-0.786583
O	-0.416924	-4.902817	2.048588
P	0.911495	-5.648371	2.839531
O	0.986696	-4.866908	4.145223
O	-2.616575	-6.200409	1.713097
O	0.525045	-7.126439	2.992494
O	2.085018	-5.417255	1.877166
O	-2.440103	7.244319	1.553407
O	-5.948830	7.463662	4.205323
H	-6.544673	1.843386	2.596606
H	-4.248402	6.469177	1.598172
H	-5.879416	6.038149	1.251217
H	-0.555659	3.940837	3.607057
H	-0.646174	1.697740	4.409200
H	-3.230830	1.307747	5.556052
H	-1.707691	-0.462083	6.559356
H	-3.874512	-0.475854	4.106798
H	-3.711388	-1.828173	5.820013
H	-0.952044	-1.294049	3.821868
H	-2.758070	-2.579979	2.633497
H	-3.181540	-1.086968	1.749593
H	-5.674149	6.923880	3.435035
H	-6.752811	7.038134	4.539461
H	-2.181024	6.465249	2.100235
H	-2.354271	8.017895	2.130740
H	0.042733	-4.072017	-1.989039
O	0.109469	-4.959778	-2.401915
H	-0.194443	-5.529753	-1.658108
H	-0.089433	-7.883736	0.224392
O	0.351080	-8.605645	0.722261
H	0.493711	-8.158918	1.597107
H	2.176709	-3.957348	0.970348
O	2.248297	-3.133638	0.420393
H	1.352838	-3.000717	0.040389

$[Mg\text{-}ATP]^{2-}$ (for valence-ionization calculations)

C	-4.047292	-1.071794	4.817318
C	-3.194817	-0.114970	4.221151
C	-2.475715	-0.525112	3.108038
N	-2.513154	-1.740345	2.542950
C	-3.346534	-2.554577	3.181839
N	-4.097823	-2.297510	4.259171
N	-2.906296	1.205666	4.524314
C	-2.038933	1.568291	3.617205
N	-1.725954	0.569169	2.731112
C	-0.823626	0.697754	1.608148
O	-1.583590	0.680709	0.409691
C	-0.873654	-0.046794	-0.596203
C	-0.141105	-1.136025	0.183624
C	0.238009	-0.414038	1.491974

O	1.509635	0.195558	1.365199
O	1.001912	-1.561784	-0.537184
C	-1.849543	-0.577298	-1.618845
O	-2.425337	0.486500	-2.385432
P	-1.934129	0.768888	-3.907146
O	-0.384407	1.198119	-3.735908
P	0.427766	2.616810	-3.422080
O	-0.545610	3.273680	-2.307929
P	-1.304772	4.770621	-2.201058
O	-0.228084	5.832729	-2.181599
N	-4.796907	-0.813912	5.897814
O	-1.961991	-0.481325	-4.730657
O	-2.775822	1.950626	-4.359405
Mg	-3.725604	3.574997	-3.794829
O	-5.503043	2.565295	-4.360801
O	-4.328064	4.919987	-5.224733
O	-2.242566	4.829846	-3.433769
O	-4.279909	3.525557	-1.819404
O	1.703808	2.182651	-2.754562
O	0.476035	3.424755	-4.688084
O	-2.125922	4.610244	-0.908624
H	-3.433038	-3.561673	2.782199
H	-4.769928	0.096850	6.362568
H	-5.378617	-1.549417	6.274898
H	-1.585350	2.547691	3.539785
H	-0.316762	1.659137	1.726105
H	0.224317	-1.080904	2.358273
H	1.997752	-0.316365	0.692341
H	-0.808943	-1.973247	0.398676
H	1.203104	-2.484079	-0.316149
H	-0.139566	0.606418	-1.083368
H	-1.339605	-1.290972	-2.273015
H	-2.673594	-1.089352	-1.115152
H	-5.341400	1.607452	-4.389346
H	-6.343071	2.685749	-3.888916
H	-5.180365	4.968544	-5.684459
H	-3.927563	5.803833	-5.234110
H	-5.115404	3.895406	-1.496983
H	-3.509291	3.976743	-1.318765
H	-3.013344	1.480029	9.793725
O	-2.922637	2.235135	9.191427
H	-3.194565	3.008622	9.710031
H	-3.780065	1.923758	6.074274
O	-4.410303	1.929844	6.828719
H	-3.873395	2.033819	7.643467

References

1. *ACD/ChemSketch*; Advanced Chemistry Development, Inc. (ACD Labs): Toronto, ON, Canada.
2. Storer, A. C., Cornish-Bowden, A., Concentration of $MgATP^{2-}$ and other ions in solution. Calculation of the true concentrations of species present in mixtures of associating ions. *Biochem. J.* **1976**, *159* (1), 1−5.
3. *Maple 2016*. Maplesoft, a division of Waterloo Maple Inc.: Waterloo, Ontario.
4. Bock, J. L., et al., ^{25}Mg NMR Studies of magnesium binding to erythrocyte constituents. *J. Inorg. Biochem.* **1991**, *44* (2), 79−87.
5. Winter, B. et al., Full Valence Band Photoemission from Liquid Water Using EUV Synchrotron Radiation. *J. Phys. Chem. A* **2004**, *108* (14), 2625−2632.
6. Weber, R. et al., Photoemission from Aqueous Alkali-Metal−Iodide Salt Solutions Using EUV Synchrotron Radiation. *J. Phys. Chem.* **2004**, *108* (15) 4729−4736.
7. Schroeder, C. Quantifying Aspects of DNA Damage. *Ph.D. dissertation*. University of Southern California: Los Angeles, CA, USA.
8. Schroeder, C. A. et al., Oxidation Half-Reaction of Aqueous Nucleosides and Nucleotides via Photoelectron Spectroscopy Augmented by ab Initio Calculations. *J. Am. Chem. Soc.* **2015**, *137* (1), 201−209.
9. Frańska, M. et al., Gas-Phase Internal Ribose Residue Loss from Mg-ATP and Mg-ADP Complexes: Experimental and Theoretical Evidence for Phosphate-Mg-Adenine Interaction. *J. Am. Soc. Mass Spectrom.* **2022**, *33* (8), 1474−1479.
